# Spatial Transcriptomics of Patients With Kaposi Sarcoma Identifies Mechanisms of Immune Evasion

**DOI:** 10.1002/jmv.70728

**Published:** 2025-12-04

**Authors:** Bahman Afsari, Ramya Ramaswami, Kathryn Lurain, Takanobu Tagawa, Daphne Knudsen‐Palmer, Guruswamy Mahesh, Ameera Mungale, Robert Yarchoan, Joseph Ziegelbauer

**Affiliations:** ^1^ HIV and AIDS Malignancy Branch, Center for Cancer Research, NCI Bethesda Maryland USA

**Keywords:** Kaposi sarcoma, KSHV, microenvironment, spatial transcriptomics, STC1

## Abstract

To identify the cell types that are infected with KSHV and the immune interactions in Kaposi sarcoma (KS) lesions, we performed spatial transcriptomics with seven KS skin tumors. We used a single‐cell RNA‐sequencing reference data set from healthy skin donors with a method to conduct spatially informed cell‐type deconvolution for spatial transcriptomics. This allowed us to predict the relative amounts of each cell type within the patient sample sections. We included custom probes for five KSHV genes that allowed us to measure human and KSHV expression patterns at the same time. We then compared the spatial gene expression data of KS skin samples with six normal skin samples and found higher expression of marker genes corresponding to macrophages/dendritic cells, lymphatic endothelial cells, and vascular endothelial cells in the KS skin lesions when compared to normal skin samples. Our spatial transcriptomic results from thousands of spots across multiple KS tumors indicated a correlation between high levels of *STC1* and decreased expression of macrophage markers. Together, these analyses offer potential mechanisms by which KSHV infection may remodel skin tissue, inhibit immune responses against KSHV infection, and confer resistance to anticancer therapies.

## 1 Introduction

The human herpesvirus 8 (HHV‐8), as known as Kaposi sarcoma herpesvirus (KSHV), is associated with a number of malignancies (reviewed in Lurain et al. [1]). These include primary effusion lymphoma (PEL), KSHV‐associated large‐cell lymphoma (KSHV‐LCL), KSHV‐associated Inflammatory Cytokine Syndrome (KICS), KSHV‐associated multicentric Castleman disease (MCD), and Kaposi sarcoma (KS). KS is one of the most common HIV‐associated malignancies which typically presents as distinct cutaneous lesions, often on the lower extremities. It can cause pain, edema, and organ‐specific symptoms depending on the extent of the disease [2]. KS is primarily driven by the expression of KSHV genes in infected spindle cells. This study is focused on skin KS lesions from clinical trial participants.

Broad questions about the microenvironment of KS have been explored in previous studies of KS in skin samples that have focused on fixed tissues and using immunohistochemistry for a subset of important human and KSHV proteins [3]. More recent studies in this field have employed bulk RNA‐sequencing [4, 5] and single‐cell RNA‐sequencing [6, 7] of KS tissue samples. Some of the unanswered questions about the microenvironment of KS include: (1) What are the differences in cell types in normal skin versus KS skin? (2) What is the immune milieu in KS lesions?

To identify the cell types that are infected with KSHV and the immune interactions in KS lesions, we performed spot‐based spatial transcriptomics with seven KS skin tumors. These methods allowed us to define 55 µm spots with or without KSHV transcripts in the same tissue section. We also identified the latent and lytic KSHV gene expression profiles in these KS skin samples. We determined a human gene panel of 75 genes whose expression in a linear combination accurately predicts whether a spot is KSHV‐infected. We used a single‐cell RNA‐sequencing reference data set from five healthy skin donors with a method to conduct spatially informed cell‐type deconvolution for spatial transcriptomics. This allowed us to predict the relative amounts of each cell type within the spots in the patient sample sections. Custom probes for five KSHV genes (ORF72, K12, PAN, ORF75, K8.1) allowed us to measure human and KSHV expression patterns simultaneously. We also discovered expression of other immune inhibitory factors at interesting locations in KS tumors. The combination of spatial gene expression patterns and predicted cell type distributions in this analysis elucidates potential mechanisms of inhibited immune responses to KSHV infections in KS lesions in people living with HIV.

When comparing human gene expression between KSHV‐infected spots and uninfected spots, one of the top differentially expressed genes in the KSHV‐infected spots was *STC1*, which we previously found to be elevated in bulk RNA‐sequencing analyses of KS tissue with matched normal tissue and we reported that levels of secreted STC1 protein increased after KSHV‐infection of primary lymphatic endothelial cells [4]. STC1 is a secreted glycoprotein that is associated with inflammation and carcinogenesis [8]. Others have previously reported that STC1 protein can inhibit the chemotactic response of macrophages to monocyte chemotactic protein and other stimuli [9]. The inverse correlation of high expression levels of STC1 and macrophage markers in the skin KS tumors, combined with previous reports of STC1 protein inhibiting macrophage chemotaxis, suggests a hypothetical model where KSHV infection of endothelial cells increases STC1 protein secretion and prevents macrophage chemotaxis to areas of KSHV infection in this model. However, changes in the spatial arrangement of cell types in KS lesions are likely the result of changing many genes, perhaps in conjunction with a gene like STC1.

## 2 Results

### 2.1 Spatial Transcriptomics Allows for Comparison of Infected and Uninfected Microenvironments

**Figure 1:**
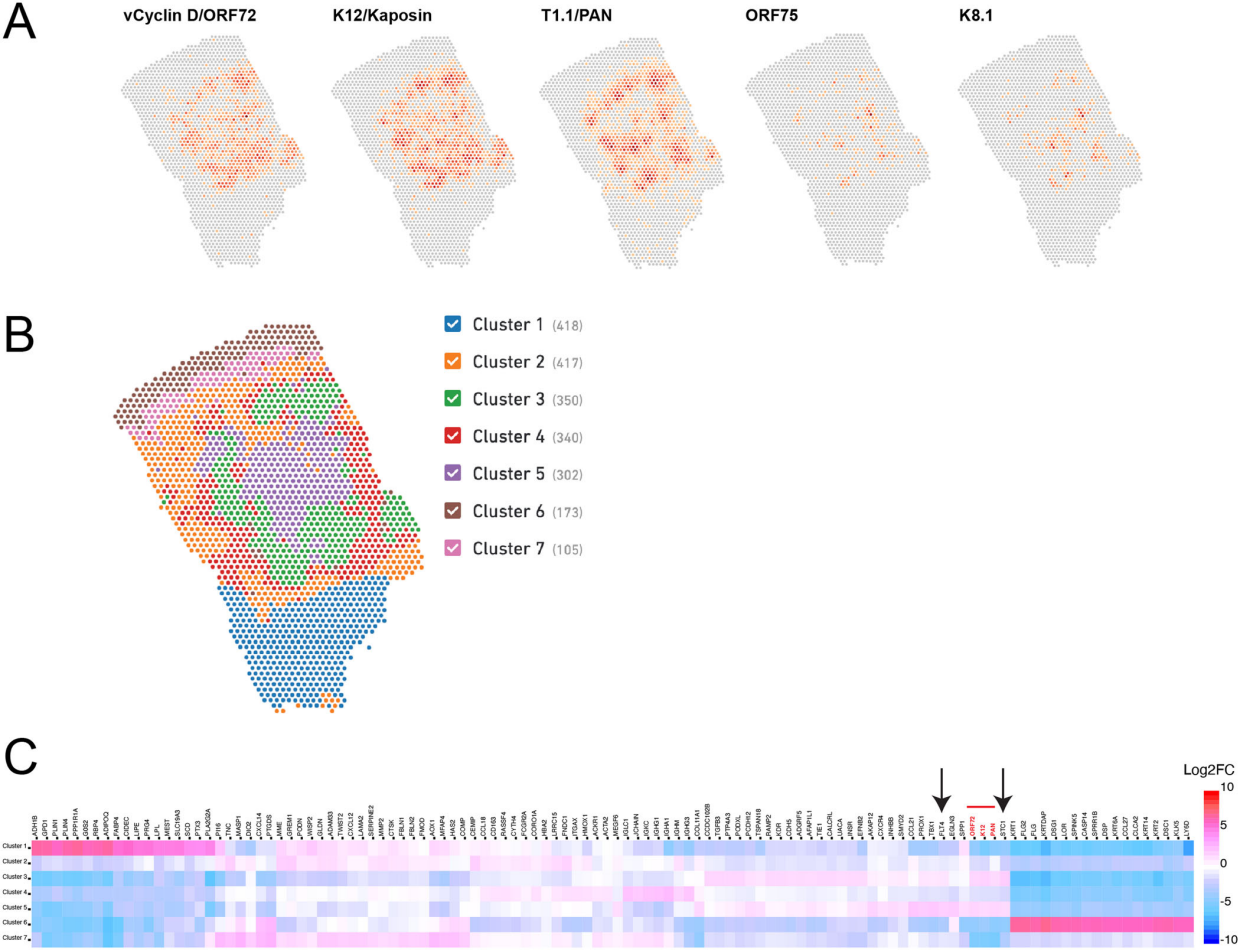
Measuring human and KSHV gene expression patterns in one KS skin tissue sample (KS_B1). (A) Spatial plots showing expression of individual KSHV genes in KS sample KS_B1. (B) Cluster analysis using human and KSHV gene expression information. Number of spots are shown next to the cluster name. (C) Heatmap showing differentially expressed genes across the seven clusters in sample KS_B1. KSHV genes are shown in red. *STC1* and *FLT4* are indicated by arrows.

The current standard for identifying KSHV infections in tissue sections is to use immunohistochemistry to detect the latent KSHV protein, LANA [10]. We have been using a new method, spatial transcriptomics [11], to investigate gene expression in KS skin sections from clinical trial participants. We detected on average 6000 genes per 55 µm spot, which corresponds to about 1–10 cells. Two sets of KS samples were processed in two separate batches. The first batch of three samples contained only information about human gene expression (KS_A1–A3). The normal spatial transcriptomics pipeline can only capture cellular transcripts, so we spiked reactions with probes for five KSHV genes in the second batch of four samples (KS_B1–B4). The same sample sections were stained with hematoxylin and eosin (Supporting Information S1: Figure 1) before processing for spatial transcriptomics. Based on previous bulk RNA sequencing and viral transcript expression patterns [4], we designed probes for the KSHV genes ORF72, K12, PAN, ORF75, and K8.1 (Figure 1A).

**Figure 2:**
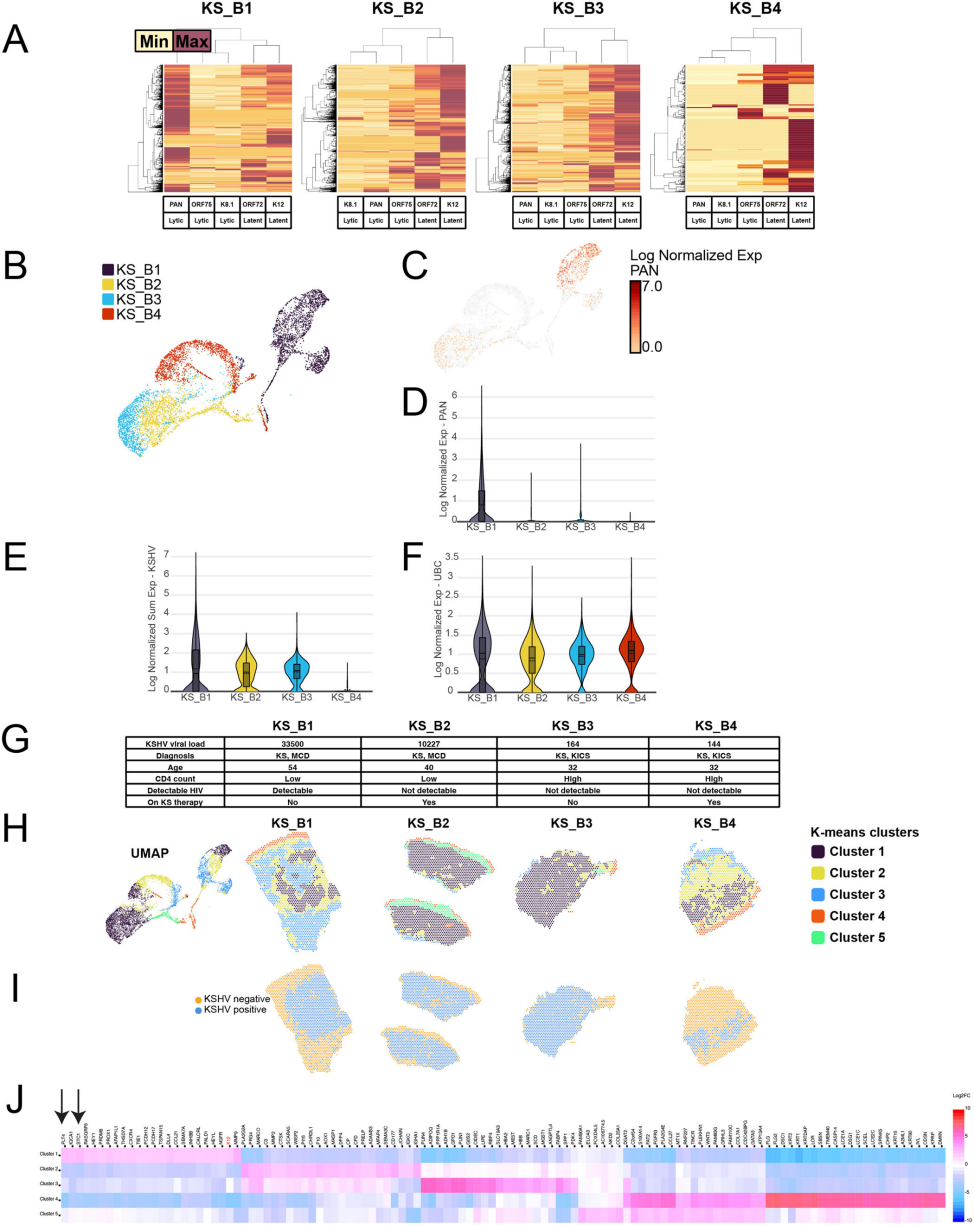
Aggregate gene expression analysis of four KS skin tissues. (A) Heatmaps showing analysis of the five KSHV genes that were measured. Each row represents an individual spot. KS_B1–B4 denote KS legions from four different patients. Higher gene expression is denoted in maroon. (B) UMAP projection of four KS samples, determined by a combination of human and KSHV gene expression. Each dot represents an individual spot of the tissue. (C) UMAP projection of the same four KS samples, but colored by KSHV *PAN* expression. (D) Violin plot of the same four KS samples, showing expression of KSHV *PAN*. (E) Violin plot of the same four KS samples, showing combined expression of five KSHV genes. (F) Violin plot of four KS samples, showing expression of a housekeeping gene, *UBC*. (G) Table denoting clinical information of the study participants that provided the tissue samples. (H) K‐means cluster analysis of these four KS samples with five clusters of overall gene expression patterns. UMAP colored by the five k‐means clusters is on the left. Spatial plot colored by these clusters is shown on the right. (I) Spatial plots displaying which spots were positive for at least one KSHV transcript. (J) Heatmap showing the differentially expressed human and KSHV genes that defined the five clusters of gene expression. KSHV gene is shown in red. *STC1* and *FLT4* are indicated by arrows.

In one of the KS tissue sections we analyzed (Figure 1B), the graph‐based clustering analysis (see Section 4 and Supporting Information) determined seven clusters based on overall human and viral gene expression patterns (clusters for samples KS_A1–A3 are shown in Supporting Information S1: Figure 2). We observed that Clusters 3 and 5 had high levels of three KSHV genes (ORF72, K12, and PAN). These are shown in the heatmap (Figure 1C). We also detected an increase within Clusters 3 and 5 in the human genes *STC1* and *FLT4*, which we found to be differentially expressed genes in our previous bulk RNA‐seq report of human KS lesions and observed a 35‐fold increase in *STC1* RNA levels upon KSHV infection in primary lymphatic endothelial cells [4].

We examined viral gene expression patterns in four KS skin lesions (KS_B1–B4, not KS_A1–A3) with these five viral genes (Figure 2A), where the heatmaps show various amounts of viral gene expression. We found that latent (ORF72 and K12) and lytic genes (K8.1, PAN, ORF75) were clustering together, suggesting that latent and lytic genes displayed similar gene expression patterns across the tissue. We also saw differences in the latent to lytic ratio, with KS sample “KS_B1” showing much more lytic gene expression than the other three samples. We also saw examples of spots that contain high amounts of a latent transcript, but little or no expression of another latent transcript. This might be a limitation of this technology and the sensitivity of these assays, since it is very likely that both latent genes (e.g., ORF72 and K12) would be expressed at the same time in the same cells in the same spots.

**Figure 3:**
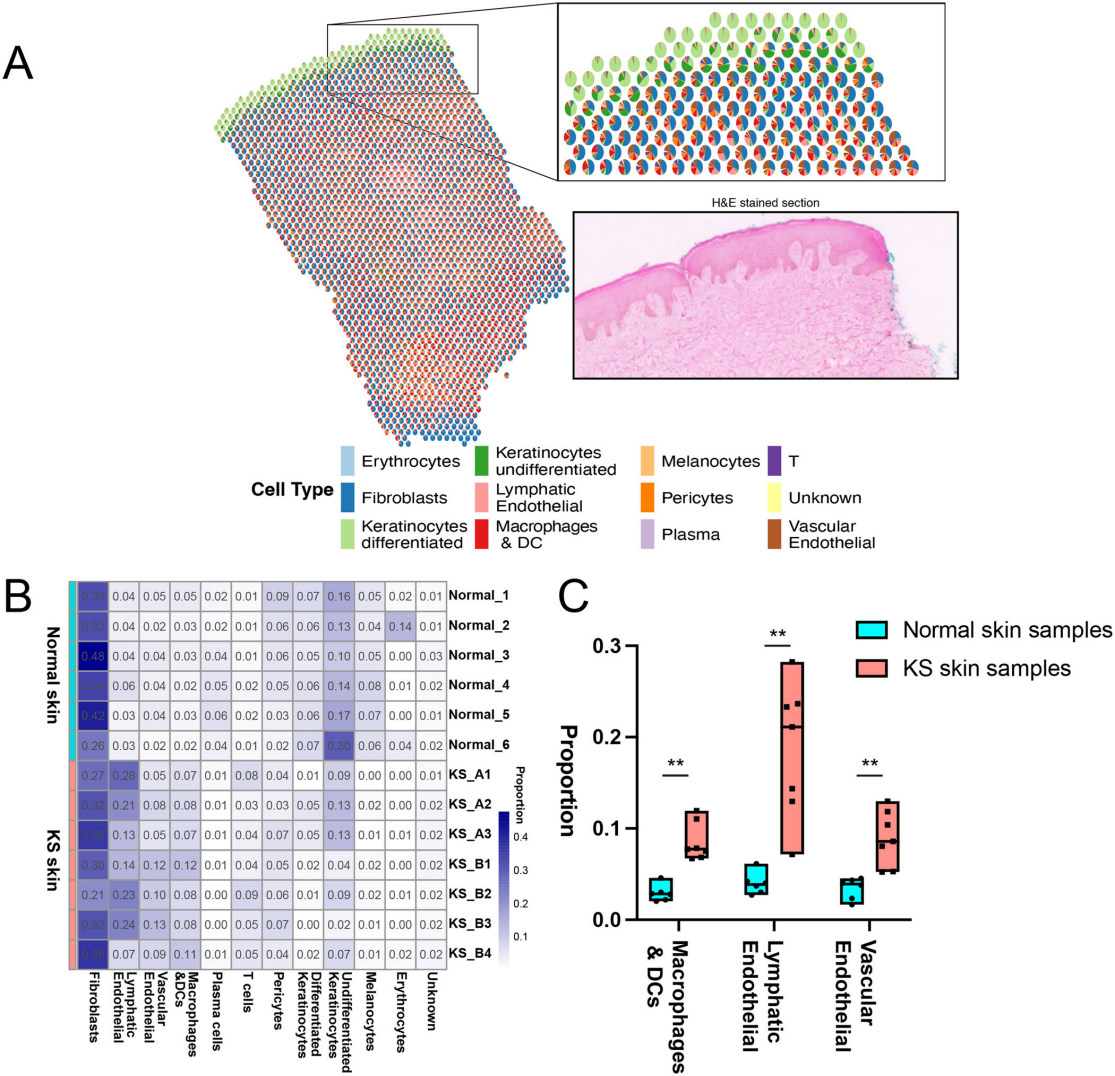
Output from spatially informed cell‐type deconvolution for spatial transcriptomics (RCTD) analysis. (A) RCTD analysis output for KS sample, KS_B1, with each spatial spot represented by a pie chart for the cell type predictions. (B) RCTD analysis was performed with data from six normal skin samples and seven KS skin samples. Average values for all of the spatial spots were calculated for each sample and shown in a heatmap. (C) A subset of cell type values from (B) is shown in box plots. ** indicates *p* < 0.01 by Mann–Whitney test.

In a separate analysis, we aggregated the four KS samples that included the KSHV gene probes (KS_B1–B4). In the UMAP graph of these four samples (Figure 2B), the sample KS_B1 was most distinct of the other three samples. The sample KS_B1 also showed more lytic gene expression when compared to the other samples (Figure 2A). This pattern of more lytic expression was again apparent when we plotted the expression of the lytic gene, PAN, across these four samples. This highest amount of PAN expression in KS_B1 is clear in both the UMAP (Figure 2C) and the violin plot (Figure 2D). Overall, the combined KSHV transcript expression with samples KS_B1, KS_B2, and KS_B3 was similar (Figure 2E), with much less KSHV expression in KS_B4. Similar expression across these four samples of a housekeeping gene, *UBC*, but variation of the KSHV gene expression suggests that there was not widespread nonspecific cross‐hybridization of the KSHV probes to human transcripts (Figure 2F). Differences in patient clinical information (Figure 2G) and small sample sizes prevented us from determining any strong correlations between expression patterns and clinical information. We optimized the hyperparameters in a separate clustering analysis using K‐means clustering (five clusters, see Section 4 and Supporting Information) to find one cluster that overlapped with the KSHV‐infected spots across multiple patient samples (Figure 2H,I). Cluster 1 overlapped with the majority of KSHV‐infected spots across samples KS_B1–B4. In the heatmap of these five clusters (Figure 2J), we found that genes like *STC1* and *FLT4* were upregulated in Cluster 1. This method is useful to find common patterns in KSHV‐infected spots across tissues. We also discovered that immunity genes like *PLA2G2A* and *CD177* genes were repressed in KSHV‐infected Cluster 1, but elevated in Cluster 2, which surrounds Cluster 1 in samples KS_B1 and KS_B2. A separate analysis using graph‐based clustering with more clusters was used to look at differences between the KS lesions (Supporting Information S1: Figure 3).

Using the four aggregated KS samples, we analyzed the number of spots that were positive for at least one transcript of five individual KSHV genes (4058 spots) and compared them to spots that were negative for KSHV transcripts (2078 spots). We queried the differentially expressed genes between KSHV‐positive spots and KSHV‐negative spots. When the genes are sorted by *p* values (Table 1), the top genes were the KSHV genes K12 and ORF72, which was partially how the two groups (infected vs. uninfected) were defined. The top differentially expressed human genes were *STC1* and *FLT4*, which is consistent with what we previously reported in KS skin tissues [4]. Other significantly altered genes include *PROX1* [12], *MMP9* [13], and *SCN9A*, which was identified in a new report using single‐cell RNA‐seq of KS tumors as increased in expression in KSHV‐infected cells [7]. These similar findings show correlations between multiple studies of KSHV infection. The functional themes in differentially expressed genes in infected spots in Table 1 include: angiogenesis and vascular development, extracellular matrix remodeling and cell adhesion, and neural development and function.

**Table 1:** Spots were separated into two groups by whether they contained zero KSHV transcripts (KSHV negative) or at least one KSHV transcript (KSHV positive).

Gene name	KSHV positive spots Log2FC	Adj. *p* value	log10 Adj. *p* value
K12	12.234	< 0.0001	Inf
ORF72	11.522	< 0.0001	Inf
STC1	2.825	< 0.0001	260.60
FLT4	2.624	< 0.0001	253.44
MMP9	3.070	< 0.0001	242.24
CEMIP	2.325	< 0.0001	197.12
IQCA1	2.593	< 0.0001	190.15
CXCR4	2.215	< 0.0001	188.70
INHBB	2.223	< 0.0001	185.49
RASGRP3	2.164	< 0.0001	177.11
AFAP1L1	2.132	< 0.0001	176.51
PROX1	2.155	< 0.0001	176.12
DLL4	2.227	< 0.0001	168.66
SCN9A	2.119	< 0.0001	166.67
PAN	12.178	< 0.0001	162.57
CCL21	2.107	< 0.0001	153.43
PCDH17	2.100	< 0.0001	153.15
PGF	1.958	< 0.0001	151.58
PRDM8	2.082	< 0.0001	150.97
PCDH12	1.972	< 0.0001	149.87
ITGA9	1.976	< 0.0001	149.01
PCSK6	1.924	< 0.0001	148.99
HEY1	2.180	< 0.0001	147.95
TSPAN15	1.952	< 0.0001	145.86
FSCN1	1.849	< 0.0001	144.35
SEMA7A	2.199	< 0.0001	143.78
EGFL7	1.808	< 0.0001	142.45
PALD1	1.934	< 0.0001	141.50
N4BP3	2.064	< 0.0001	138.62
BCL6B	1.884	< 0.0001	137.45

*Note:* Differentially expressed genes were determined across these two groups and the top genes are shown, sorted by adjusted *p* values.

**Figure 4:**
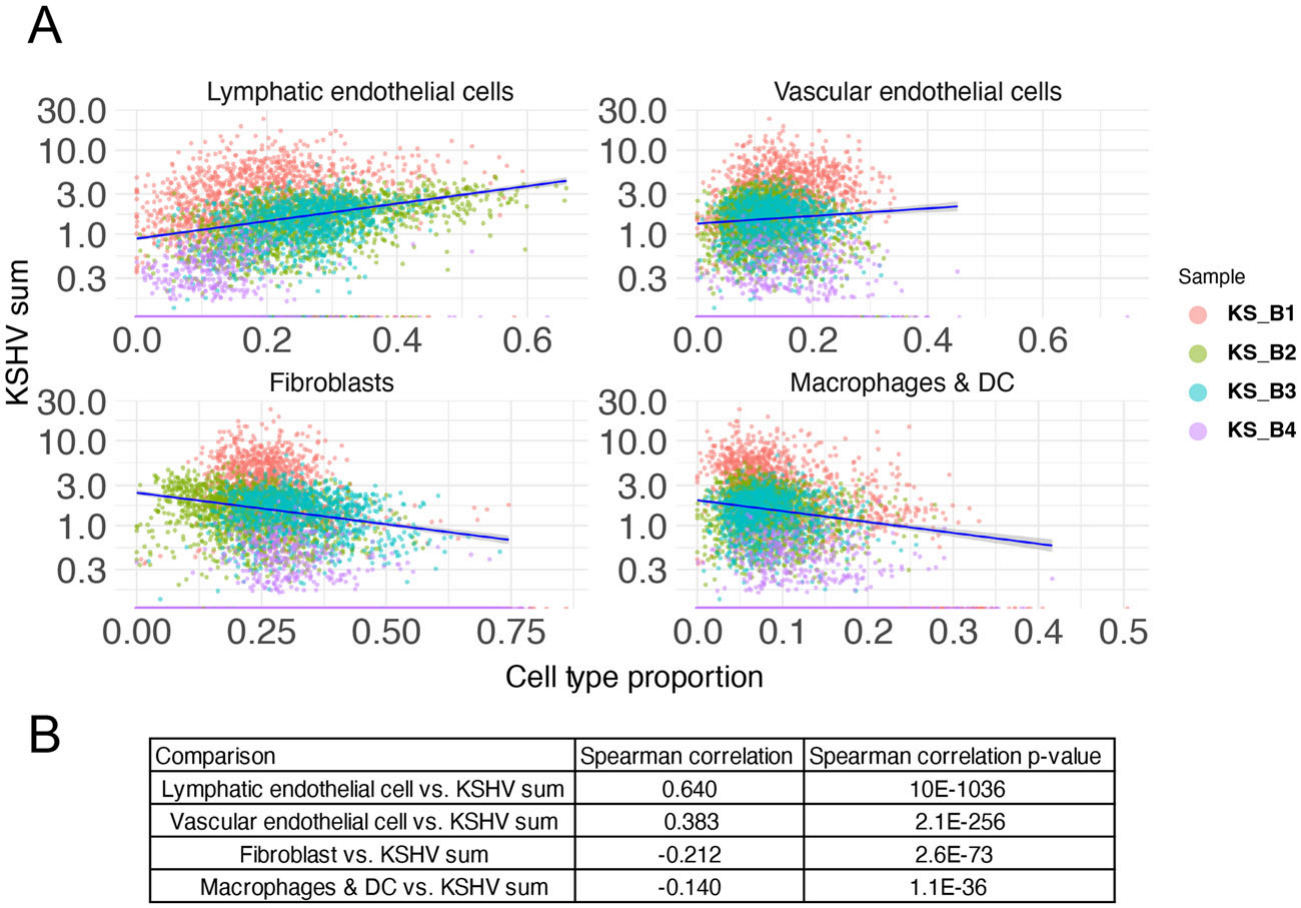
Combining total KSHV gene expression and cell type prediction values from RCTD analysis. (A) Correlations between total KSHV gene expression (KSHV sum) and RCTD‐based cell type prediction proportions are shown for a subset of cell types. (B) Spearman's correlation values and *p* values are shown in the table.

We next asked whether a human gene expression signature could predict KSHV‐infected spots. We trained an algorithm (see Supporting Information) based on a comparison of the top 1000 differentially expressed genes when comparing infected versus uninfected spots, with multiple tissue samples and then tested the prediction algorithm on an untrained sample. After multiple rounds of testing with four KS samples and six normal skin samples, we developed a high‐accuracy prediction model with an area under the curve equal to 0.96 (Supporting Information S1: Figure 4A). This prediction model contains 75 cellular genes, 73 of which were upregulated in the KSHV‐infected spots compared to uninfected spots. The two downregulated genes were *CXCL14* and *CFD*, which are both immune response genes. We sorted the 75 genes based on their significance in the prediction algorithm (Supporting Information S1: Figure 4B). The most significant gene in predicting KSHV‐infected spots was *HMOX1*, which has been shown to be regulated and a number of previous genomic studies looking at gene expression changes after KSHV infection [14, 15]. *STC1* was the fourth gene in our current list and *FLT4* was the 13th gene, consistent with our previous findings. This model could be used by others to predict viral infection without having to use custom KSHV probes for viral transcript detection.

### 2.2 Analysis of Predicted Cell Types in Tissue Microenvironments

**Figure 5:**
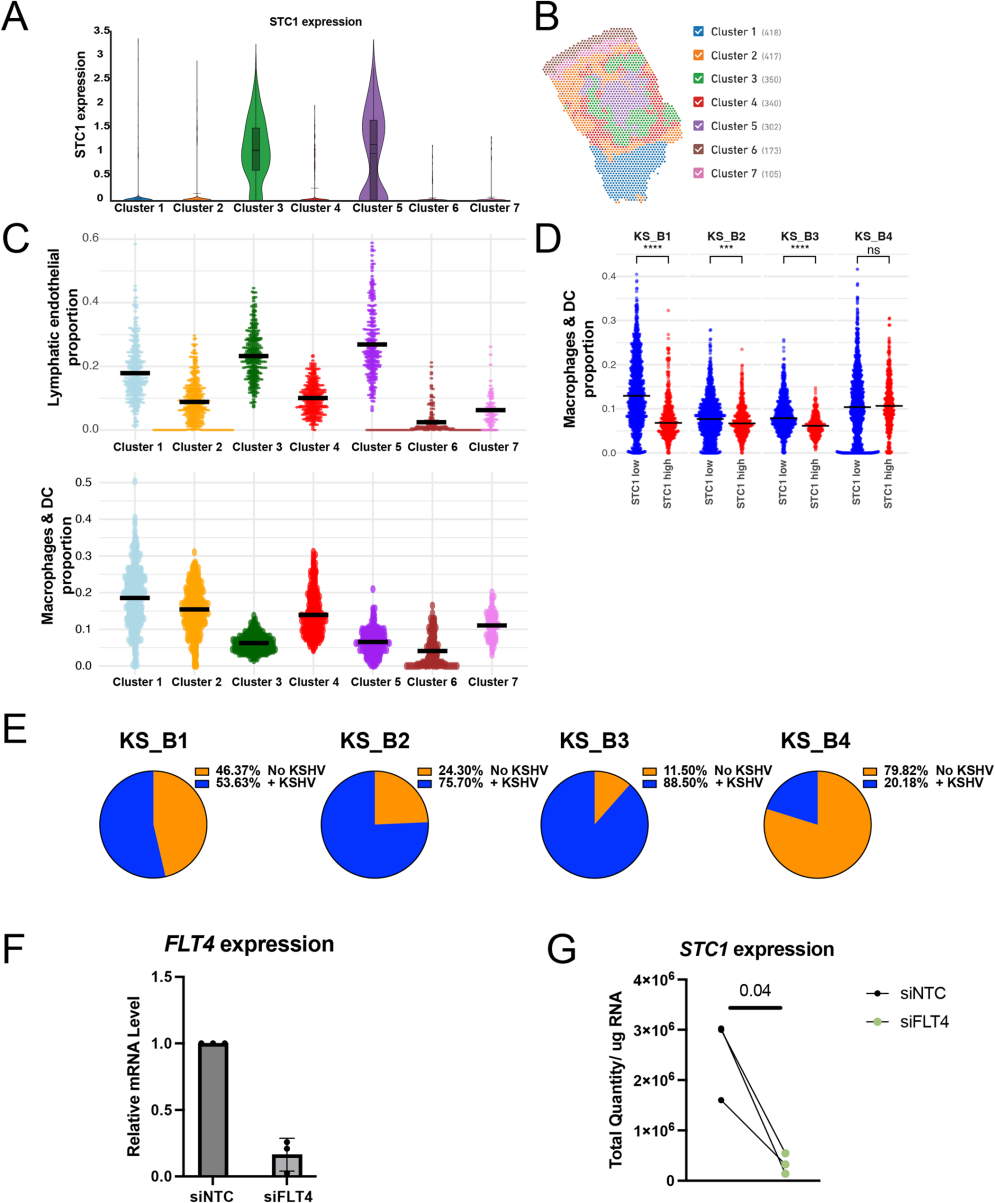
Expression patterns of *STC1* and macrophage & dendritic cell markers. (A) Violin plot of *STC1* expression in the seven clusters depicted in Figure 1. (B) Seven gene expression clusters in KS sample, KS_B1, are shown. (C) RCTD‐based cell‐type prediction proportions for lymphatic endothelial cells, macrophages, and dendritic cells for the seven clusters are plotted. (D) Spots for four KS samples were separated into either STC1 low or STC1 high groups. RCTD‐based cell‐type prediction proportions for macrophages and dendritic cells are plotted on the vertical axis. Mann–Whitney test was used to test for significance, **** indicates *p* < 0.0001 and *** indicates *p* < 0.001. (E) Pie charts depict the portion of spots that had zero KSHV transcripts (orange) or were positive for KSHV transcripts (blue). (F) Repression of *FLT4* using siRNAs with transcript measurements by RT‐qPCR. SiNTC is a nontargeting control. (G) Primary lymphatic endothelial cells were depleted of *FLT4* using siRNAs. *STC1* transcripts were measured using RT‐qPCR.

Since this spatial transcriptomic platform did not provide single‐cell resolution, we wanted to query the different cell types that were represented in the spots, which contain multiple cells and can be of different cell types. To make predictions of the various cell types represented in each spot, we used a method for spatially informed cell‐type deconvolution for spatial transcriptomics (called RCTD algorithm) [16]. This method requires a relevant single‐cell RNA‐seq reference set. We used a normal skin reference set [17] that included data from 15 000 cells from 5 donors. This reference set contained 11 different cell types (identified by differentially expressed genes). Using this cell‐type reference set, we applied the RCTD algorithm to our spatial data sets, which estimated portions of various cell types per spot (Figure 3A). One positive control was to investigate the epidermal regions on tissue sections. As shown in Figure 3A, the top layer of the epidermis contained spots with gene expression patterns indicative of differentiated keratinocytes (symbolized in light green). Just below that layer, we observed a majority of cells were estimated to be undifferentiated keratinocytes. These predicted cell type patterns follow the known pattern of keratinocytes in the epidermis and the matching H&E staining pattern (Figure 3A), which gave us confidence in this method to proceed with further analysis. When we combined the RCTD analysis and the K‐means cluster analysis (Supporting Information S1: Figure 5), we observed the K‐means Cluster 4 was enriched for keratinocyte markers.

Our initial analysis of cell types in these KS tissues started at the sample level, where we used the averaged values for all of the spots (taking the RCTD relative proportion values for each spot and averaging the values for all spots in sample) to look at cell‐type distributions across samples. We used spatial transcriptomics and RCTD analysis output data from six normal skin samples from a previous report [18] and our seven KS skin samples. As expected, we found the majority of cell types in the normal skin samples to be fibroblasts (Figure 3B). Looking beyond fibroblasts, we found increased expression of markers for macrophages and dendritic cells, lymphatic endothelial cells, and vascular endothelial cells in KS skin samples (Figure 3C).

We sought to understand whether more lymphatic or vascular endothelial cell markers correlated with the level of KSHV expression in individual spots [19, 20, 21]. We analyzed all of the spots in four KS samples and found a strong correlation (*r* = 0.64, *p* < 10e − 1036) between the total amount of KSHV gene expression and lymphatic endothelial marker expression (Figure 4A). There was a correlation with vascular endothelial marker expression but less significant (*r* = 0.38, *p* = 2e − 246) (Figure 4B) and negative correlation with fibroblast marker expression (*r* = −0.21, *p* = 2e − 73) (Figure 4B). This suggests that where there was higher KSHV gene expression, there were more lymphatic cells than vascular endothelial cells. There could be multiple biological reasons (e.g., tropism, chemotaxis, proliferation) for this correlation, but this current data set cannot strongly distinguish between these multiple potential mechanisms. We found a negative correlation between total KSHV gene expression and macrophage markers (*r* = −0.14, *p* = 1e − 36) (Figure 4B). Taken together, we found more macrophage and DC marker expression in the KS samples compared to normal skin samples (Figure 3C), but less expression of these markers in the spots that contained high KSHV expression (Figure 4A). These observations present the hypothesis that macrophages might be attracted to KS lesions, but cannot migrate into the areas of high KSHV infection and expression.

Previous reports have shown that STC1 can inhibit macrophage chemotaxis and chemokinesis [9]. We used the graph‐based clustering (see Section 4 and Supporting Information) approach to distinguish different clusters of spots in sample KS_B1 (first shown in Figure 1B). These clusters are defined by overall gene expression patterns using human and viral genes. In the sample KS_B1, Clusters 3 and 5 had the highest amounts of *STC1* expression (Figure 5A,B) and viral gene expression (Figure 1C). Clusters 3 and 5 also had lower expression of marker genes for macrophages and dendritic cells (Figure 5C). There was a negative correlation for *STC1* expression and macrophage markers in six of the seven clusters. Cluster 6 was the only outlier, but Cluster 6 contains the epidermis, which is dominated by keratinocytes. In summary, clusters of spots outside of the epidermis displayed a negative correlation between *STC1* expression and macrophage/dendritic cell gene expression.

We expanded this analysis of *STC1* and macrophage markers to include all four KS samples with viral transcript data. We separated spots into two groups: (a) with log‐normalized expression of *STC1* < 1.0, and (b) with *STC1* expression higher than 1.0. In three of the four KS samples, we observed a significant decrease in the macrophage markers in the spots with high expression of STC1 (Figure 5D). The fourth sample (KS_B4) did not show this trend, but this sample also had a minority of infected spots (Figure 5E). Thus, in the samples with a majority of KSHV‐infected spots, we observed a negative correlation of *STC1* expression and macrophage markers.

Vascular endothelial growth factor (VEGF) signaling has been shown to promote *STC1* expression [22]. We tested whether repression of *FLT4/VEGFR3* (Figure 5F) would alter *STC1* expression. We found siRNAs that targeted *FLT4* resulted in lower *STC1* expression in primary lymphatic endothelial cells (Figure 5G).

**Figure 6:**
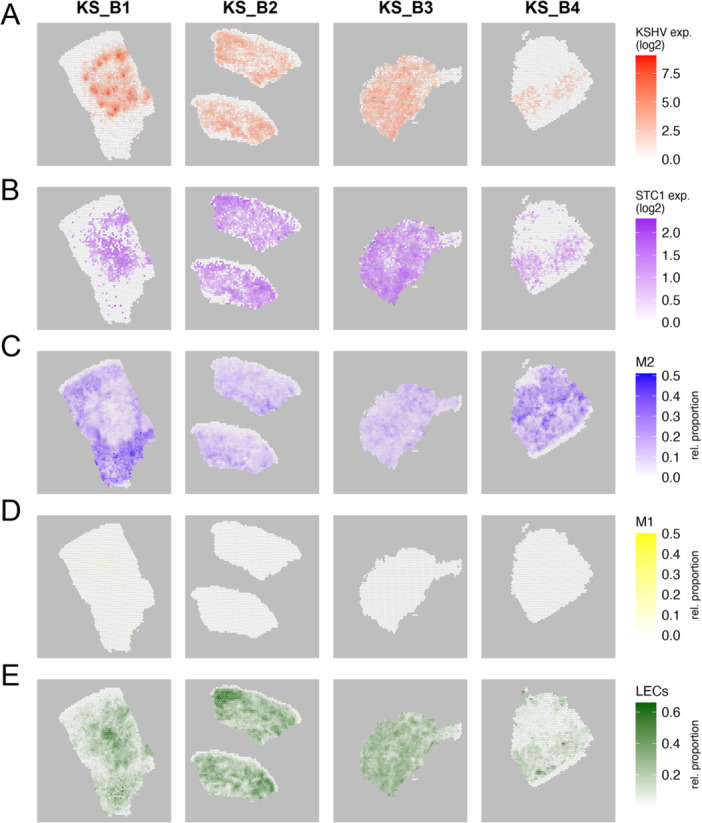
Spatial patterns of KSHV, *STC1*, and macrophage marker (M1, M2) expression. (A) Combined expression of KSHV genes is shown in spatial plots. (B) Expression of *STC1* is shown in spatial plots. (C) RCTD analysis of relative proportions of M2 macrophage markers is shown. (D) RCTD analysis of relative proportions of M1 macrophage markers is shown. (E) RCTD analysis of relative proportion of lymphatic endothelial cells is shown.

We then investigated the spatial expression patterns of KSHV genes, *STC1*, and macrophage (M1/M2) marker genes. We found a strong correlation in the spatial patterns of KSHV gene expression and *STC1* across the four samples (Figure 6A,B). In areas of lower expression of KSHV and *STC1* expression, we observed higher expression of M2 macrophage markers surrounding high levels of KSHV expression (Figure 6C). This trend was more pronounced in samples KS_B1 and KS_B4 and note that these samples had some larger areas with lower *STC1* expression. We detected little expression of M1 macrophage in these samples (Figure 6D). We used ~1000 differentially expressed genes (of which almost 700 were used by the RCTD algorithm) to distinguish M2 and M1 gene expression (Supporting Information S2: Table 1). Moreover, the areas with high KSHV and STC1 expression show high LEC marker gene expression (Figure 6E). Immunohistochemistry of sections from the same tissue blocks for lymphatic cells (LYVE1) and M2 macrophages (CD163) shows correlative widespread expression of these marker proteins (Supporting Information S1: Figure 6).

We also used an alternative method to RCTD, called CARD [23], and included these in Supporting Information S1: Figures 8–16. Most of the general conclusions in this cell type deconvolution remained unchanged when using either method, although RCTD appears to generate cell decomposition results that are more consistent with the lymphatic endothelial results at the sample‐level (Figure 3B) and the individual spot‐level (Figure 4A).

### 2.3 Immune Cells Surrounding KSHV‐Infected Areas

**Figure 7:**
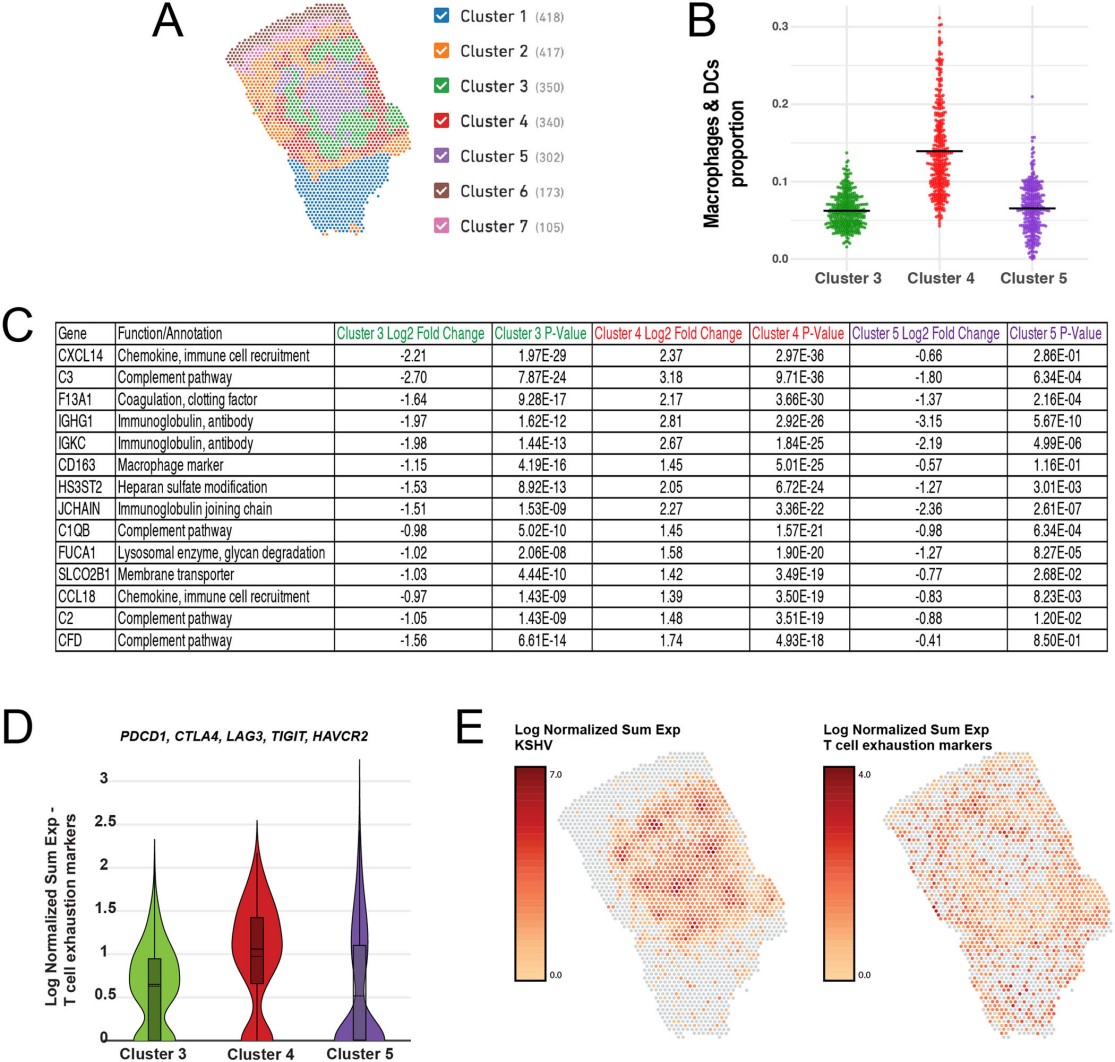
Markers of immune suppression surround KSHV‐infected areas. (A) Seven gene expression clusters in KS sample, KS_B1, are shown. (B) RCTD‐based cell‐type prediction proportions for macrophages and dendritic cells are shown for KSHV‐infected Clusters (3 and 5) and the surrounding Cluster 4. (C) Genes that were differentially expressed when comparing Clusters 3, 4, and 5. Shown are genes that were upregulated in Cluster 4 and sorted by *p* value. (D) A violin plot shows the expression of combined T‐cell exhaustion markers (*PDCD1, CTLA4, LAG3, TIGIT, HAVCR2*) in the seven clusters of KS sample, KS_B1. (E) Spatial plots showing the expression of the combined KSHV genes or combined T‐cell exhaustion markers.

In one KS sample (KS_B1), there was roughly an equal number of infected and uninfected spots. We dove deeper into spatial gene expression patterns in this sample for this reason. In this sample, Clusters 3 and 5 were infected (Figure 1C, graph‐based clustering). Cluster 4 surrounded Clusters 3 and 5 (Figure 7A), so we looked at differentially expressed genes among Clusters 3, 4, and 5. We found that *CCL21* was the highest upregulated gene in Cluster 5 (Supporting Information S3: Table 2). *CCL21* is secreted by endothelial cells and has been shown to be a chemokine for macrophages and T cells [24], a host response pathway. However, in Cluster 3, we found high expression of *CALCRL*, which inhibits macrophages and dendritic cells from presenting antigens to T cells [25]. Surrounding the infected Clusters 3 and 5 was Cluster 4, which appears to contain many immune cells, including macrophages (Figure 7B,C). The evidence for macrophages in Cluster 4 (Figure 7B) came from the RCTD analysis of hundreds of genes (Figure 5C) and some of the specific macrophage‐related genes (*CD163*, *C3*) in Figure 7C. We found high expression of *C3* in Cluster 4, which is part of the complement system (important for bridging innate and adaptive immune responses). Furthermore, the highest differentially expressed gene in Cluster 4 was *CXCL14*, which inhibits angiogenesis and recruits NK, DC, and T cells [26]. We analyzed the combined expression of a panel of T cell exhaustion markers (*PDCD1, CTLA4, LAG3, TIGIT, HAVCR2*) and found the highest expression in Cluster 4 (Figure 7D), suggesting that most of the exhausted T cells are in Cluster 4. In the spatial plots of combined KSHV expression or T cell exhaustion markers, we observed that the highest levels of T cell exhaustion markers were outside of the regions of high KSHV expression (Figure 7E).

## 3 Discussion

To our knowledge, this study represents one of the first reports of using spatial transcriptomics with KS patient samples using custom probes for multiple KSHV genes. These assays bring light to the spatial gene expression patterns of both human and viral genes. It is important to note the current limitations of the spatial transcriptomics platform used in this report. For using fixed tissue sections, we were forced to use probe‐based transcript detection. This prevents the detection of novel transcripts and is only able to query transcripts in the predetermined set of probes. Low transcript read counts were common and raises the strong possibility of false negatives in the analysis. Lack of observation of a specific transcript in an individual spot could mean that the transcript was expressed poorly, but simply not detected. This reality urged our analysis away from trying to define latent versus lytically infected spots, since we did not feel confident about filtering out false negative spots. Additionally, this analysis was not at the single‐cell resolution and the heterogeneity of the patient samples and small sample size of seven KS samples should be considered limitations of this study.

Our previous bulk RNA‐sequencing analysis [4] and this report have discovered interesting connections between STC1 in KSHV infections in KS lesions. A number of publications about STC1 have interesting connections to KS biology. STC1 has been shown to promote tumor cell viability and proliferation [27]. Higher *STC1* expression in tumor tissues (e.g., ESCC) was correlated with decreased overall survival in humans [28]. VEGF treatment stimulates *STC1* transcription [22] and STC1 promotes the expression of VEGF [29]. These facts are important in the context of increased angiogenesis in KS lesions [30]. We also showed that the inhibition of STC1 using siRNAs led to lymphatic endothelial tubule formation to be decreased [4]. In this report, we demonstrated that repression of FLT4/VEGFR3 decreased *STC1* expression (Figure 5G). STC1 protein has been shown to be a secreted glycoprotein that has roles in the regulation of phagocytosis and macrophages (review Wang et al. [31]). In our previous report [4], we showed that upon infection of primary lymphatic endothelial cells the levels of secreted STC1 protein increased by ELISA. Additionally, a recent report identified the receptor of STC1 as IGFR2 [32] and their results showed that treatment of macrophages with STC1 protein inhibited IL‐1β secretion from the macrophages, thus inhibiting an inflammatory response. Taken together, our hypothetical model (Supporting Information S1: Figure 7) is that upon infection of endothelial cells with KSHV, endothelial cells secrete more STC1 protein. This increased secreted STC1 protein could inhibit macrophage chemotaxis to infected cells and also may inhibit IL‐1β secretion from macrophages to inhibit a pro‐inflammatory response to infection. We also note that the observations about macrophage location within the tissues are likely due to many gene expression changes and not the result of a single altered gene.

IL‐1β is a key protein in the pro‐inflammatory response to infections [33]. M2 macrophages are generally thought to be anti‐inflammatory and immunosuppressive [34]. We also found increased M2 macrophages surrounding KSHV‐infected areas (Figures 6 and 7). Additionally, we observed expression of T‐cell exhaustion markers adjacent to KSHV‐infected areas (Figure 7D,E). We propose a hypothetical model that decreased IL‐1β (perhaps from STC1 effects), increased M2 macrophages, and exhausted T cells would appear to promote an overall immunosuppressive environment for KSHV‐infected cells to flourish. Utilizing methods to inhibit immunosuppressive factors could help promote a more robust immune response to KSHV infections and likely better immunosurveillance and cancer control. This is particularly important as a potential mechanism of treatment resistance to anti‐PD‐1 therapies, which have been found to be effective in KS [35].

## 4 Methods

### 4.1 Patient Cohort and Specimen Collection

Individuals with KS under the care of the HIV/AIDS Malignancy Branch at the National Cancer Institute were included and had a 6 mm punch biopsy of cutaneous KS. All of these lesions were nodular lesions and all participants included in the study had T1 stage KS. KS biopsies were divided, and a portion was sent to the laboratory of pathology for histological confirmation of KS by LANA IHC. All participants were consented to protocols for tissue procurement (NCT00006518) and/or sequencing of KS and other KSHV‐associated diseases (NCT03300830) to permit RNA sequencing of KS lesions for this study. Both protocols were approved by the NCI Institutional Review Board. All enrolled participants gave written informed consent in accordance with the Declaration of Helsinki.

### 4.2 Spatial Transcriptomics

FFPE skin sections were cut and placed on slides. Tissue sections were stained with H&E and used the Spatial 3′ v1 or v2 chemistry Visium platform (10X Genomics). Custom KSHV probes were designed and used according to Technical Note CG000621 (10X Genomics). Research reported in this publication was supported by the University of Michigan Advanced Genomics Core, the UM Single Cell Spatial Analysis Program, and the National Cancer Institutes of Health under Award Number P30CA046592 by the following Cancer Center Shared Resource: Single Cell and Spatial Analysis Shared Resource. Library prep and next‐generation sequencing were carried out in the Advanced Genomics Core at the University of Michigan. Analysis was performed using Space Ranger (10X Genomics), Loupe Browser (10X Genomics), and Seurat [36]. Raw sequencing data are available at GSE300380. We used 10X Genomics graph‐based clustering and K‐means clustering (see Supporting Information for further details). When comparing samples together (KS_B1–B4), we used the “aggr” pipeline of Space Ranger.

### 4.3 Spot Prediction Model

We used a logistic regression model to develop a predictor for infection in a spot (based on human gene expression). We define a spot infected or KS positive if at least one read from KSHV has been identified in that spot; otherwise, we call the spot uninfected or KS negative. First, we applied the standard Seurat package (v 5.1.0) pipeline: merged all samples, normalized data, scaled data, and found 2000 most variable genes using FindVariableFeatures with default parameters (“vst”). We found the genes whose expression *t*‐test effect size is more than medium (|Cohen's *D*| > 0.5) among the top variable genes. After that, we used a logistic regression model with the Lasso penalty (cv.glmnet with parameter family = “binomial” from glmnet package “4.1.8” with a default value which returns a chosen value for parameter “lambda”). We applied the trained logistic regression model (with the chosen “lambda” in the training) to the test spots. We ran a ten‐fold cross‐validation over all four KS‐positive and six Normal samples to validate our methodology. We calculated the average of the area under the ROC curve over the test spots to measure accuracy. As a representative predictor, we applied our gene selection process to all the spots and calculated the Logistic Regression coefficients. We defined the importance score of each gene in the predictor as its coefficient multiplied by the difference of average gene expression between KS‐positive and KS‐negative spots.

### 4.4 Cell Type Decomposition Analysis

We employed two methods, RCTD and CARD. We obtained the cell composition by using RCTD in the package spacer (1.0.0) [16] and the CARD package (CARD_1.1) [23]. To do the cell decomposition, both RCTD and CARD require a single‐cell transcriptome reference with cell‐type annotations; we provided RCTD and CARD with five normal samples from the skin (GSE130973), which include cell‐type annotations. We passed the raw read counts (not normalized) of the spatial data to both of the RCTD and CARD algorithms as required by the algorithms. RCTD and CARD assigned portions for each spot to 11 cell types plus an “unknown” cell type category. Since the cell reference lacks specific immune cell subtypes, we used another cell reference (GSE214695) to estimate the portion of subtypes of immune cells. This reference has two sub‐cell types. We used the coarser cell‐types Nanostring annotation, which divided macrophages/DCs into five subcategories: M0, M1, M2, IDA macrophages, and DCs.

### 4.5 siRNA‐Mediated Transcript Depletion

Primary human dermal lymphatic endothelial cells (Promocell #C‐12216) were incubated at 37°C with 20 nM ON‐TARGETplus siRNA targeting FLT4 (Horizon #L‐003138‐00‐0005) and a nontargeting control (Horizon #D‐001810‐10‐05) for 24 h using DharmaFECT1 Transfection Reagent (Horizon #T‐2001‐03). After 24 h, transfection media was replaced with EGM2.

### 4.6 RNA Extraction and RT‐qPCR

Total RNA was extracted using Direct‐zol RNA Miniprep Kit (Zymo Research #R2053) with on‐column DNase I digestion. For cDNA generation, 0.5 μg RNA was used with ReverTra Ace qPCR RT master mix (Toyobo #FSQ‐101). qPCR was performed with Thunderbird Next SYBR qPCR mix (Toyobo #QPX‐201) and THUNDERBIRD Next Probe qPCR Mix (Toyobo #QPC‐101) on the StepOnePlus real‐time PCR system (ThermoFisher).

## Author Contributions

Bahman Afsari, Ramya Ramaswami, Kathryn Lurain, and Joseph Ziegelbauer contributed to the conception and design of the project. Bahman Afsari, Ramya Ramaswami, Kathryn Lurain, Takanobu Tagawa, Daphne Knudsen‐Palmer, Robert Yarchoan, and Joseph Ziegelbauer wrote, edited, and revised the manuscript. Bahman Afsari, Ramya Ramaswami, Kathryn Lurain, Takanobu Tagawa, Daphne Knudsen‐Palmer, Guruswamy Mahesh, Ameera Mungale, Robert Yarchoan, and Joseph Ziegelbauer contributed to sample acquisition, analysis, and interpretation. Joseph Ziegelbauer supervised the project.

## Conflicts of Interest

R. Yarchoan, R. Ramaswami, and K. Lurain report receiving research support from Celgene (now Bristol Myers Squibb), CTI BioPharma (a Sobi A.B. Company), PDS Biotech, and Janssen Pharmaceuticals through CRADAs with the NCI. R. Yarchoan, R. Ramaswami, and K. Lurain also report receiving drugs for clinical trials from Merck, EMD‐Serono, and Eli Lilly and preclinical material from Lentigen Technology through CRADAs or MTAs with the NCI. R. Yarchoan is a coinventor on US Patent 10 001 483 entitled “Methods for the treatment of Kaposi's sarcoma or KSHV‐induced lymphoma using immunomodulatory compounds and uses of biomarkers.” An immediate family member of R. Yarchoan is a coinventor on patents or patent applications related to internalization of target receptors, epigenetic analysis, and ephrin tyrosine kinase inhibitors. All rights, title, and interest to these patents have been assigned to the US Department of Health and Human Services; the government conveys a portion of the royalties it receives to its employee inventors under the Federal Technology Transfer Act of 1986 (P.L. 99‐502).

## Supporting information

Supplemental information_submit.

Table S1 M1 vs. M2 genes.

Table S2 KS B1 Cluster 3, 4, 5, sorted by 5FC.

## Data Availability

The data that support the findings of this study are openly available in NCBI GEO at https://www.ncbi.nlm.nih.gov/geo/, reference number GSE300380. All additional data are available in the Supporting Files and NCBI GEO GSE300380.
