# Supplementary material for: Spatial Transcriptomics of Patients With Kaposi Sarcoma Identifies Mechanisms of Immune Evasion
Source: J Med Virol. 2025 Dec 4;97(12):e70728. doi: 10.1002/jmv.70728 (PMC12676656; doi:10.1002/jmv.70728)
Supplement: Supplementary file 1 — Supplemental information_submit. [file JMV-97-e70728-s002.docx]

Supplemental Material

**Figures**

Figure S1


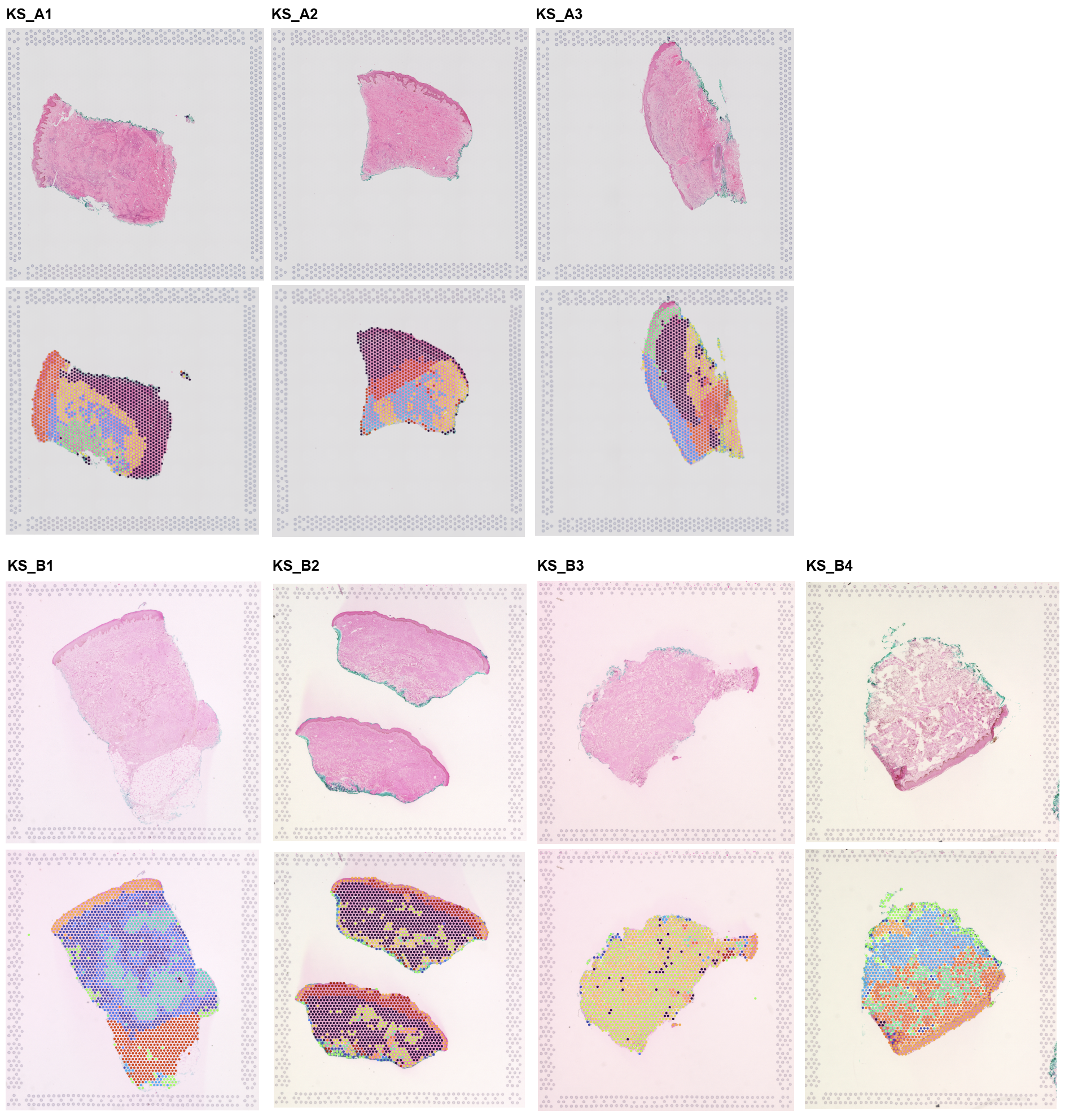


Figure S1. Hematoxylin and eosin staining of the same seven KS skin lesions as used for spatial transcriptomics. Overlay of spatial transcriptomics spots are shown in the bottom rows. Colors of spots correspond to graph-based clustering with legends shown in Figures S2 and S3.

Figure S2

Figure S2. Graph-based clustering of KS samples KS_A1 to KS_A3.

S2A. Spatial plots of the clusters for each independent sample cluster analysis.

S2B. Heatmaps showing the genes used to define the separate clusters. Colors show log2 fold change values when comparing gene expression to the rest of the clusters.

Figure S3


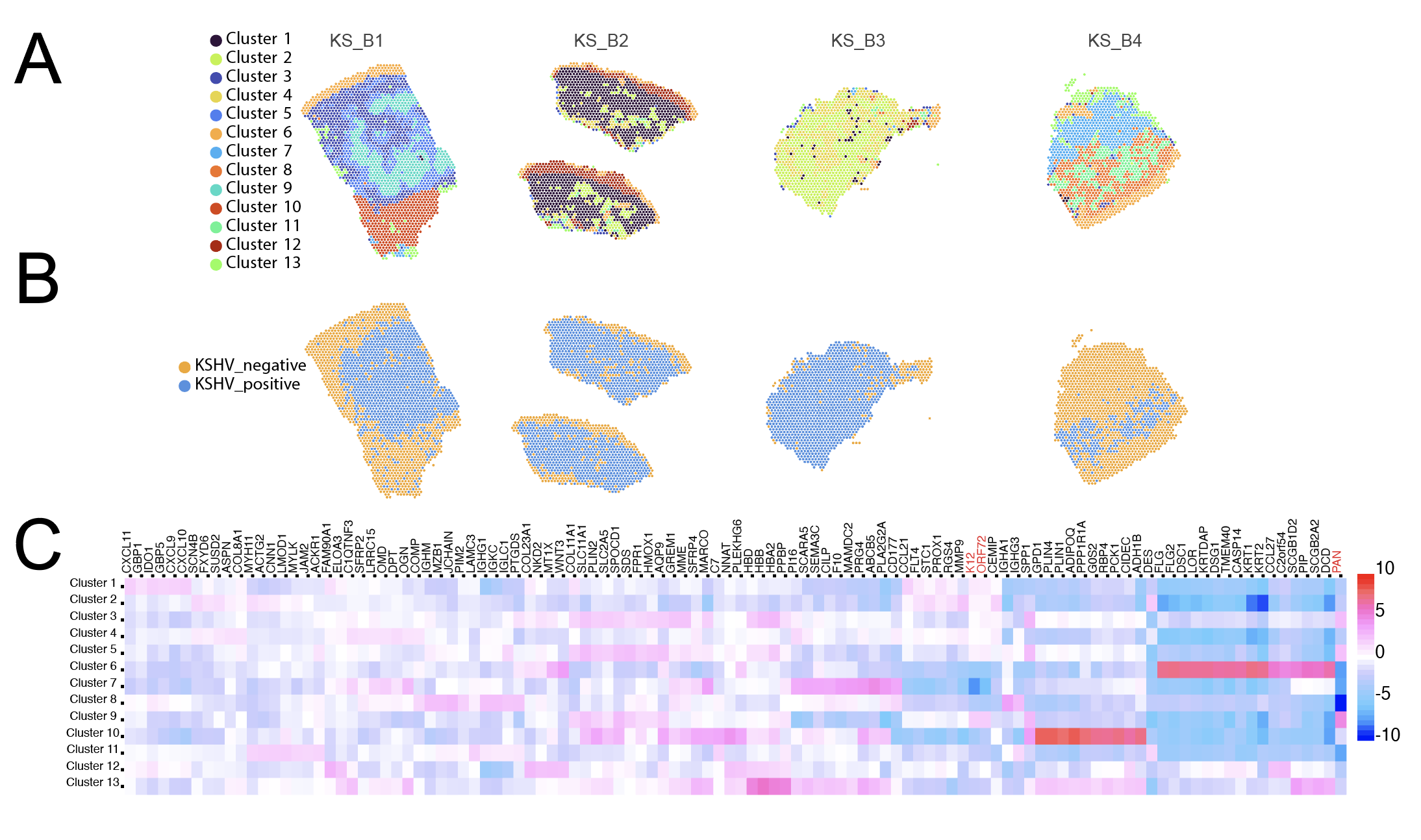


Figure S3. Graph-based cluster analysis of 4 aggregated samples.

3A. Cluster analysis of these four KS samples found 13 clusters of overall gene expression patterns.

3B. Spatial plots displaying which spots were positive for at least one KSHV transcript.

3C. Heatmap showing the differentially expressed human and KSHV genes that defined the 13 clusters of gene expression. KSHV genes are shown in red.

Figure S4


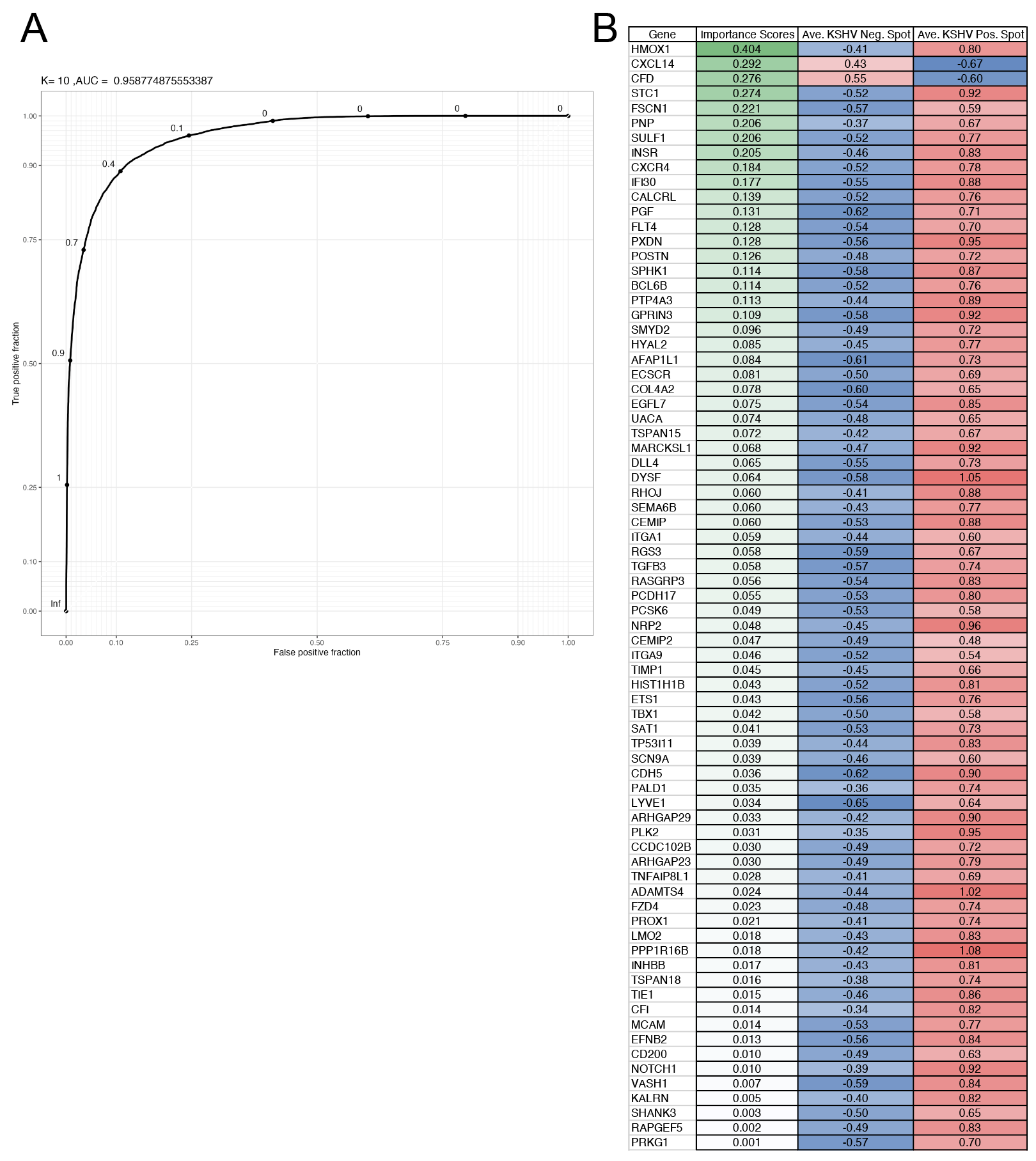


Figure S4. Prediction model for estimating if a given spot is KSHV-infected, based on human gene expression pattern.

4A. Receiver operating characteristic (ROC) curve depicting the accuracy of the prediction algorithm. The area under the curve (AUC) value is shown above the graph.

4B. Specific genes that are used by the prediction model are shown and sorted by their importance value to the prediction algorithm. The third and fourth columns show the average log-normalized expression of each gene in KSHV-negative and KSHV-positive spots. Blue colors denote lower expression and red values show higher expression.

Figure S5


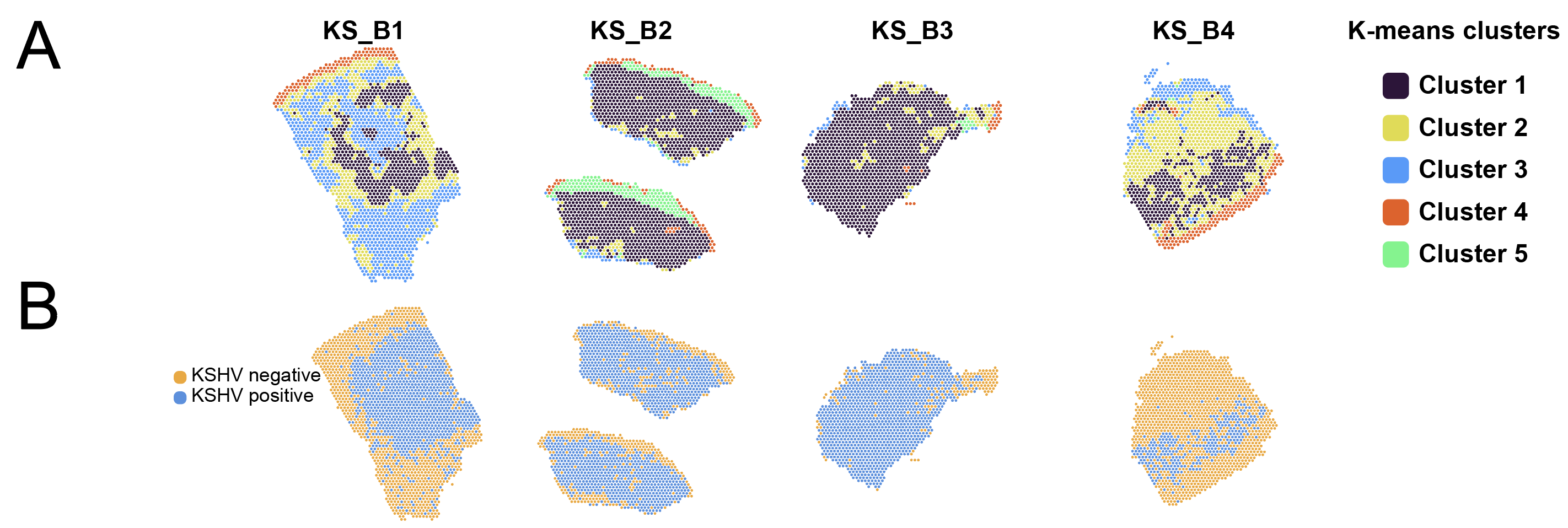


C


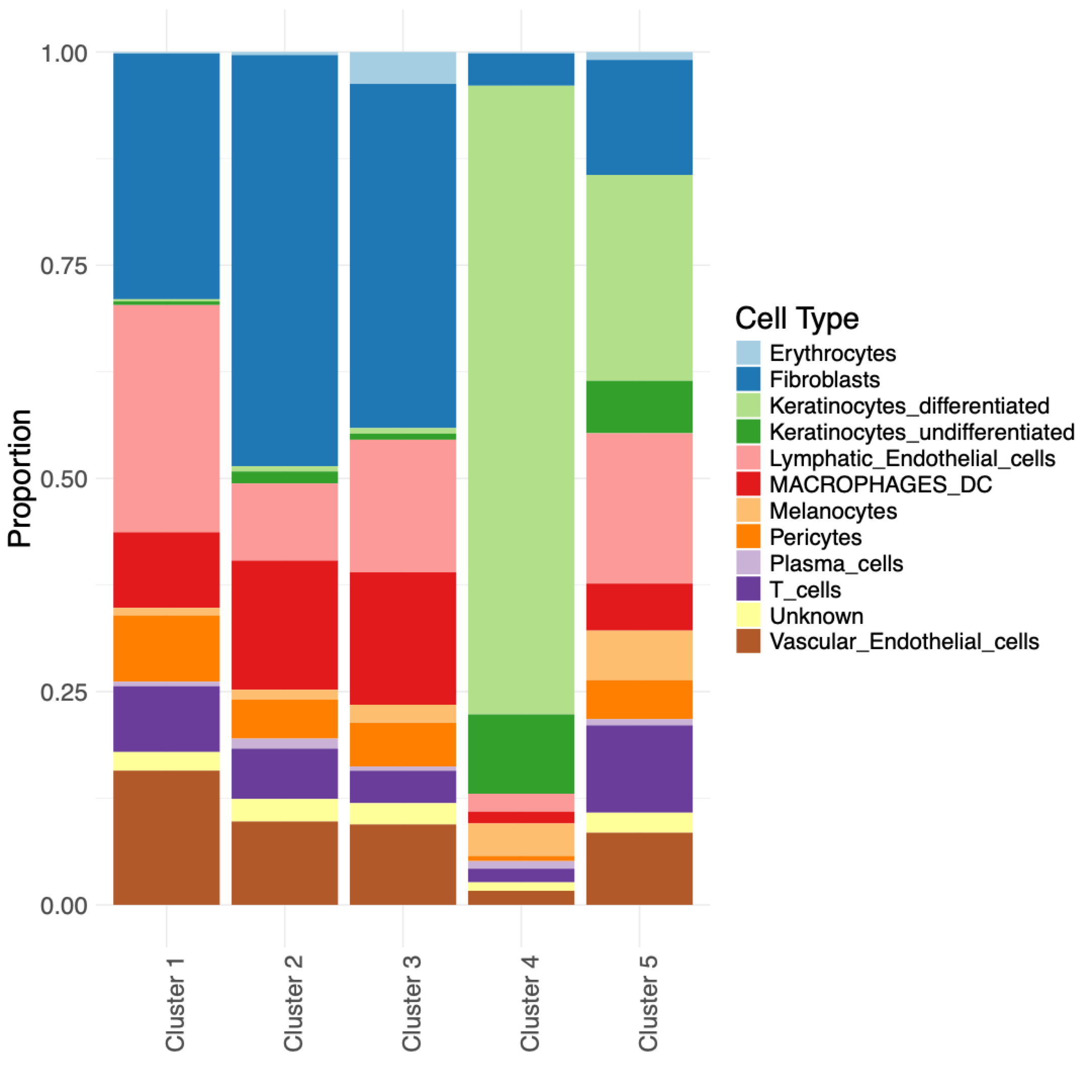


Figure S5. Integration of K-means clustering and RCTD cell type predictions.

5A-B. The same spatial plots from Fig. 2.

5C. Relative proportions of predicted cell types from RCTD analysis with the five K-means clusters.

Figure S6


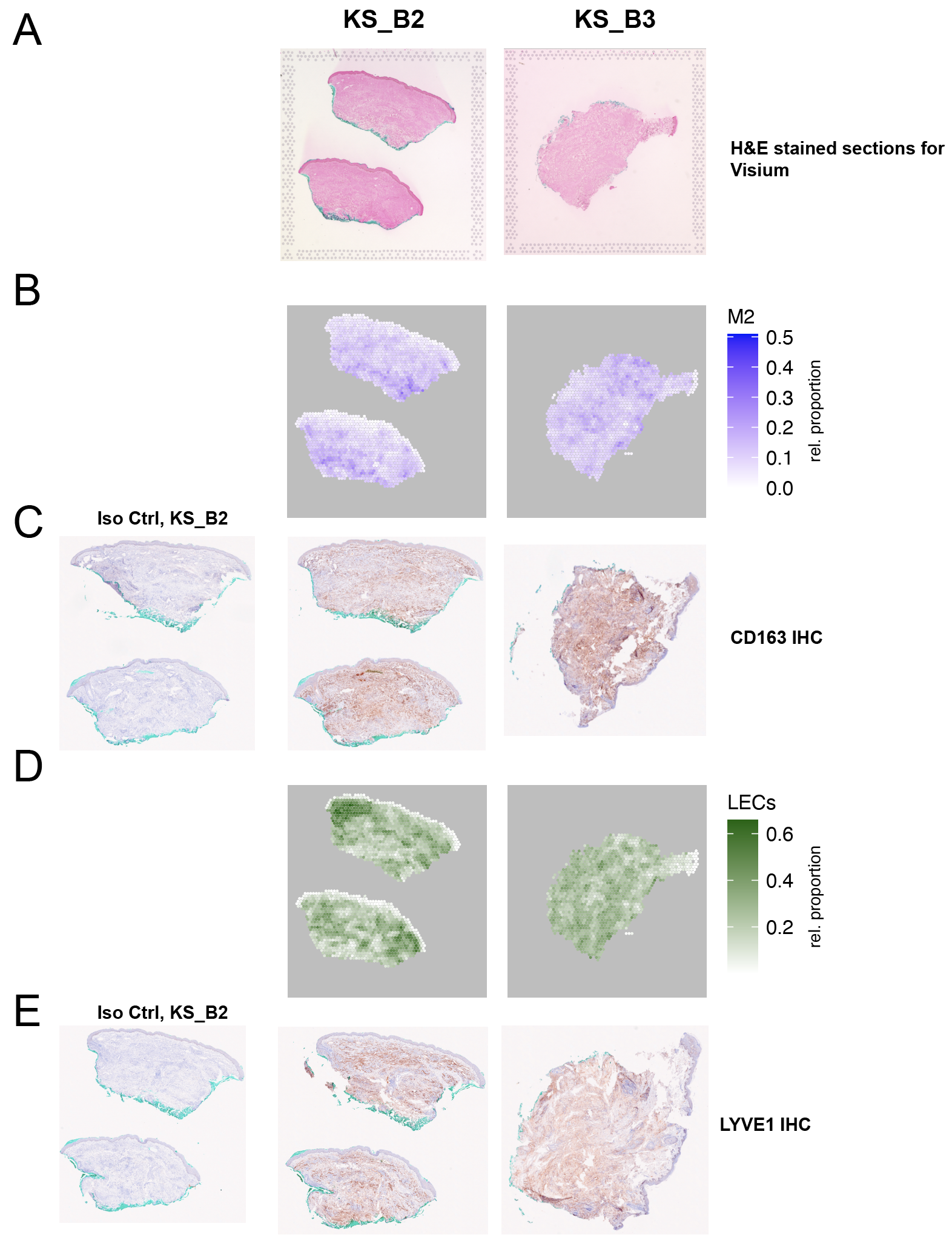


Figure S6. Immunohistochemistry for sections for markers of M2 macrophages (CD163, Novocastra #NCL-L-CD163) and lymphatic endothelial cells (LYVE1, Abcam #ab219556).

S6A. H&E stained sections that were used for Visium spatial transcriptomics.

S6B. M2 macrophages proportion predictions for Visium sections using RCTD program.

S6C. Different sections from the same tissue blocks, but stained with antibody isotype control (left) or with CD163 antibody.

S6D. Lymphatic endothelial cell proportion predictions for Visium sections using RCTD program.

S6E. Different sections from the same tissue blocks, but stained with antibody isotype control (left) or with LYVE1 antibody.

Figure S7


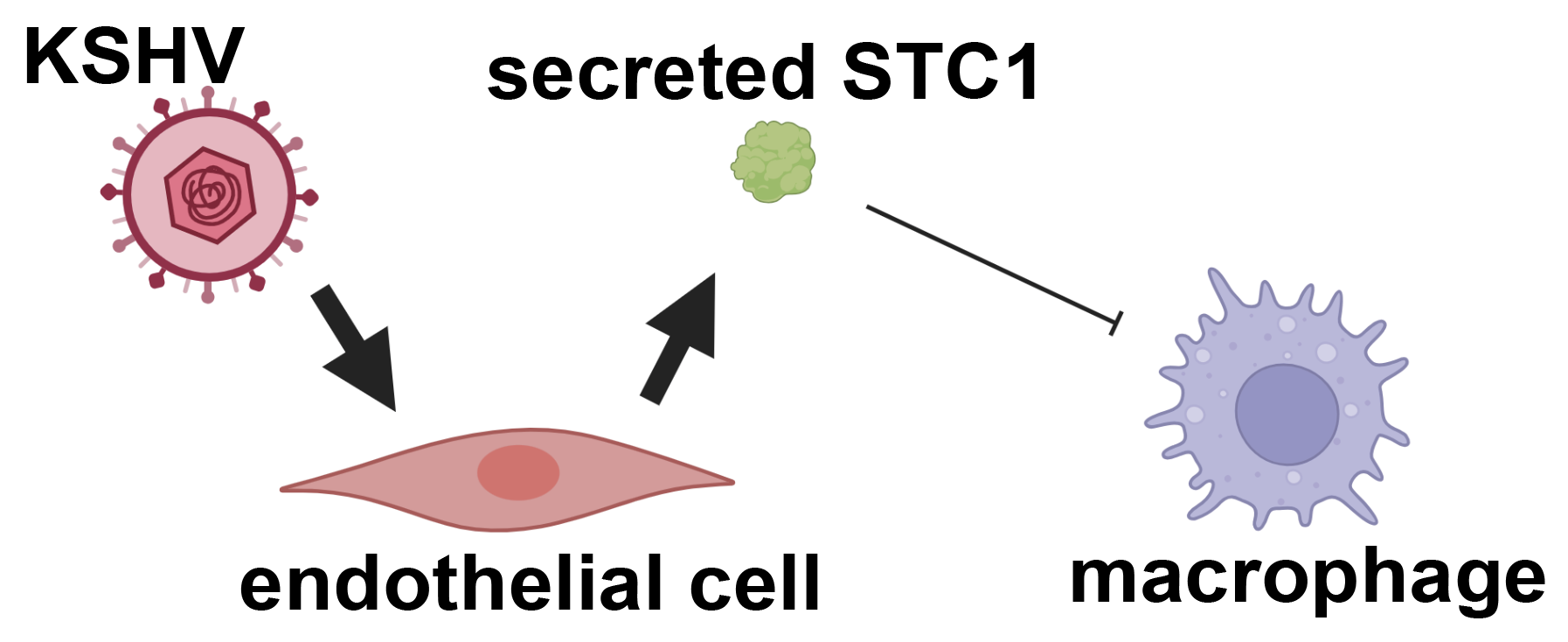


Figure S7. Hypothetical model based on spatial transcriptomics and previous literature information. The combined information presents a hypothetical model that KSHV infection of endothelial cells increases secretion of STC1 protein. Then STC1 protein can inhibit macrophage chemotaxis and function.

Figure S8


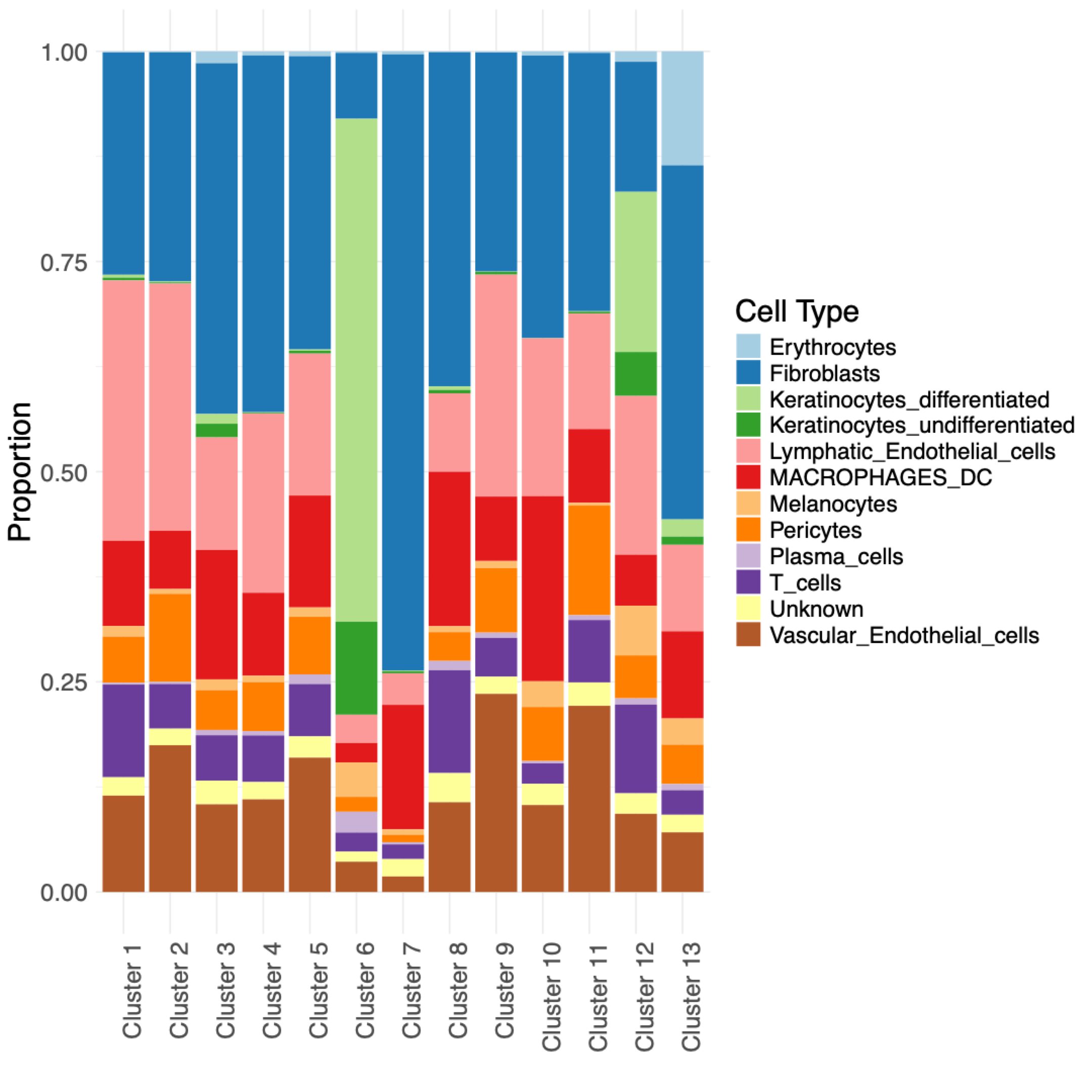


Figure S8. Relative proportions of predicted cell types from RCTD analysis with the Graph-based clusters. The clustering was performed simultaneously for all four samples, as shown in Figure S3A. Cluster six represents primarily the epidermis.

Figure S9


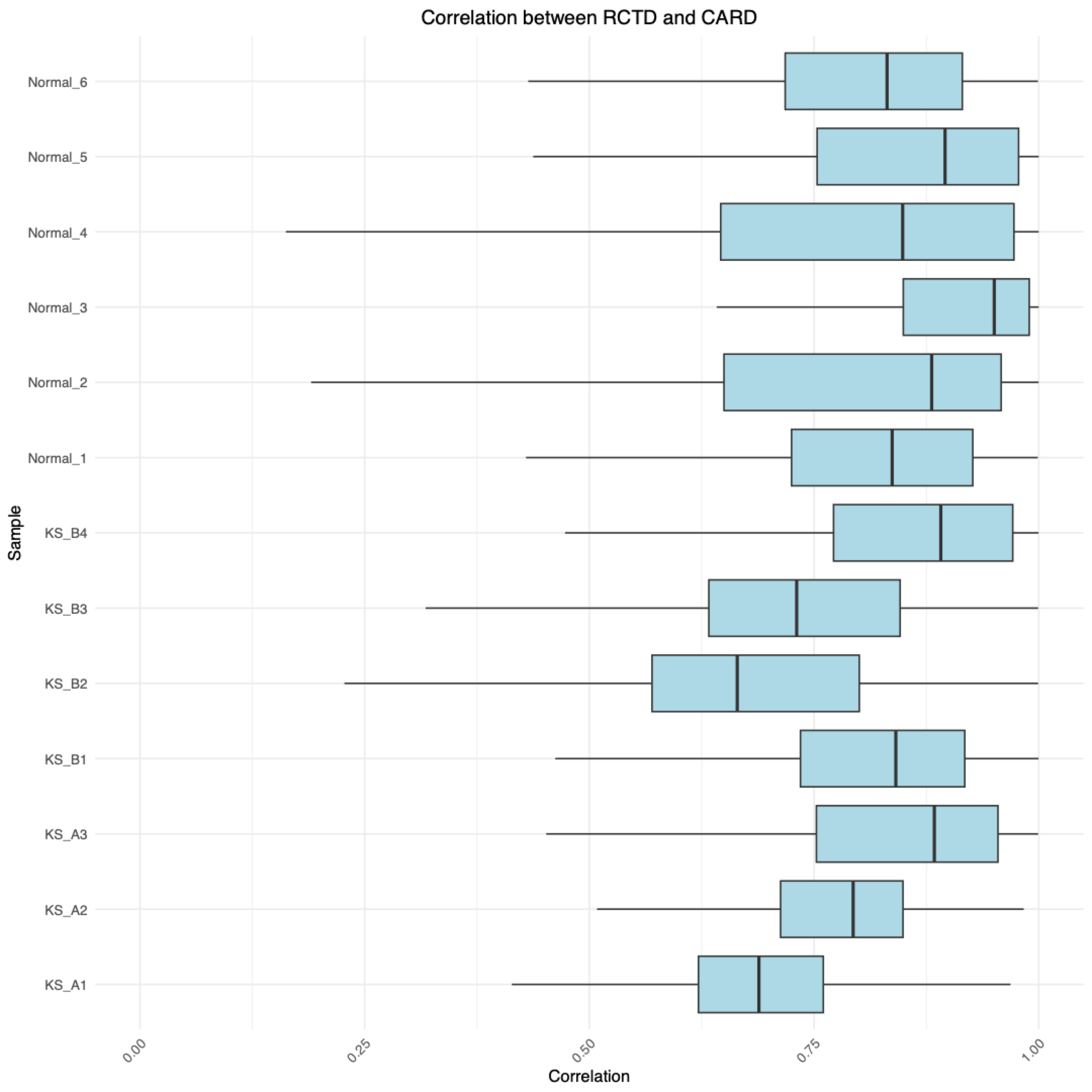


Figure S9. Comparison of CARD and RCTD: The boxplot of the correlation of proportions between CARD and RCTD for each spot in a sample. Overall, the correlation is high, however, some samples have a lower correlation.

Figure S10


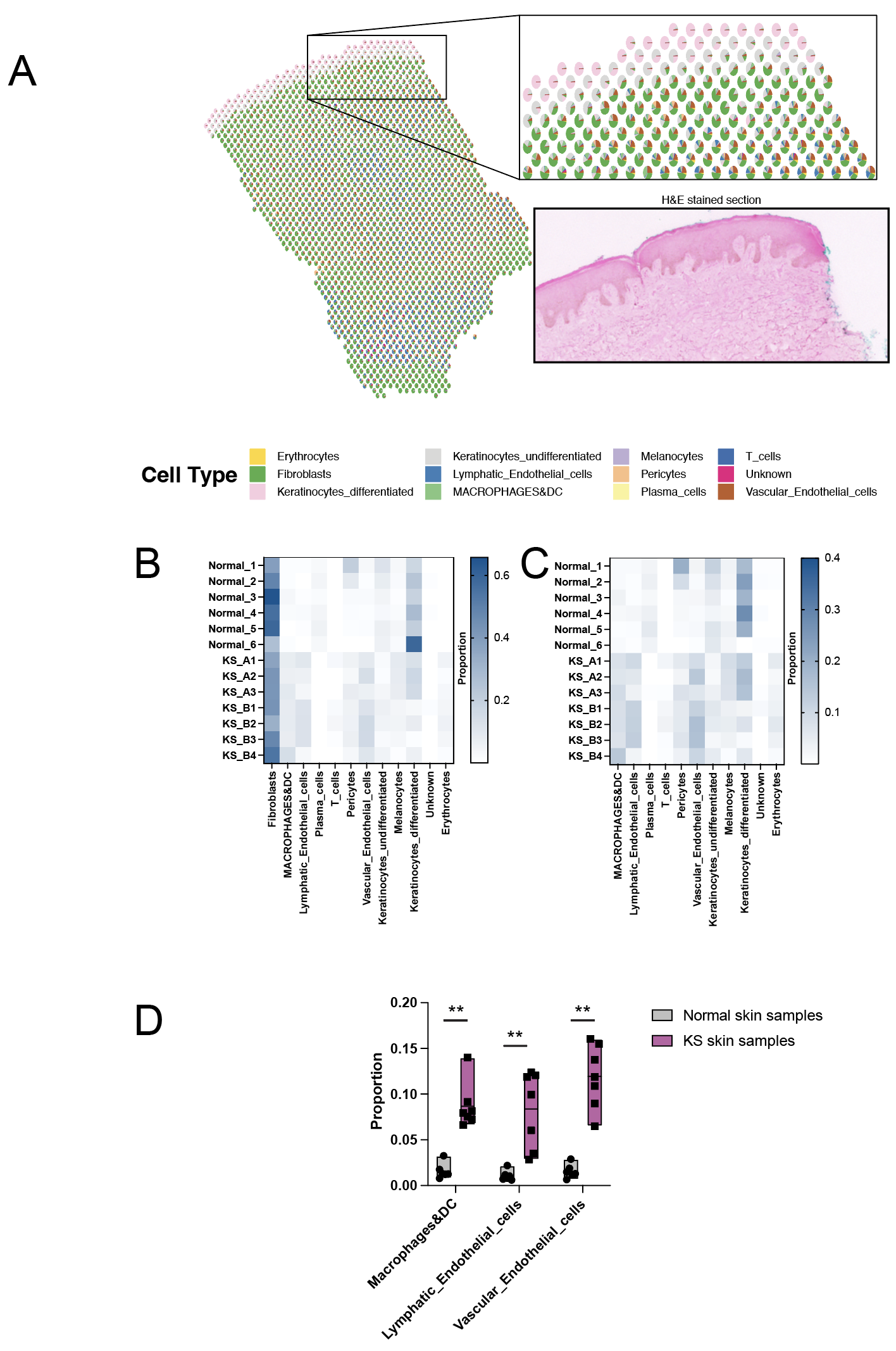


Figure S10. Output from spatially informed cell-type deconvolution for spatial transcriptomics (CARD) analysis.

S10A. CARD analysis output for KS sample, KS_B1, with each spatial spot represented by a pie chart for the cell type predictions.

S10B. CARD analysis was performed with data from six normal skin samples and seven KS skin samples. Average values for all of the spatial spots were calculated for each sample and shown in a heatmap.

S10C. The same information as in 4B, but with the fibroblast category removed and the color bar focused on lower values.

S10D. A subset of cell types values from 4B are shown in box plots. ** indicates p<0.01 by Mann Whitney test.

Figure S11.


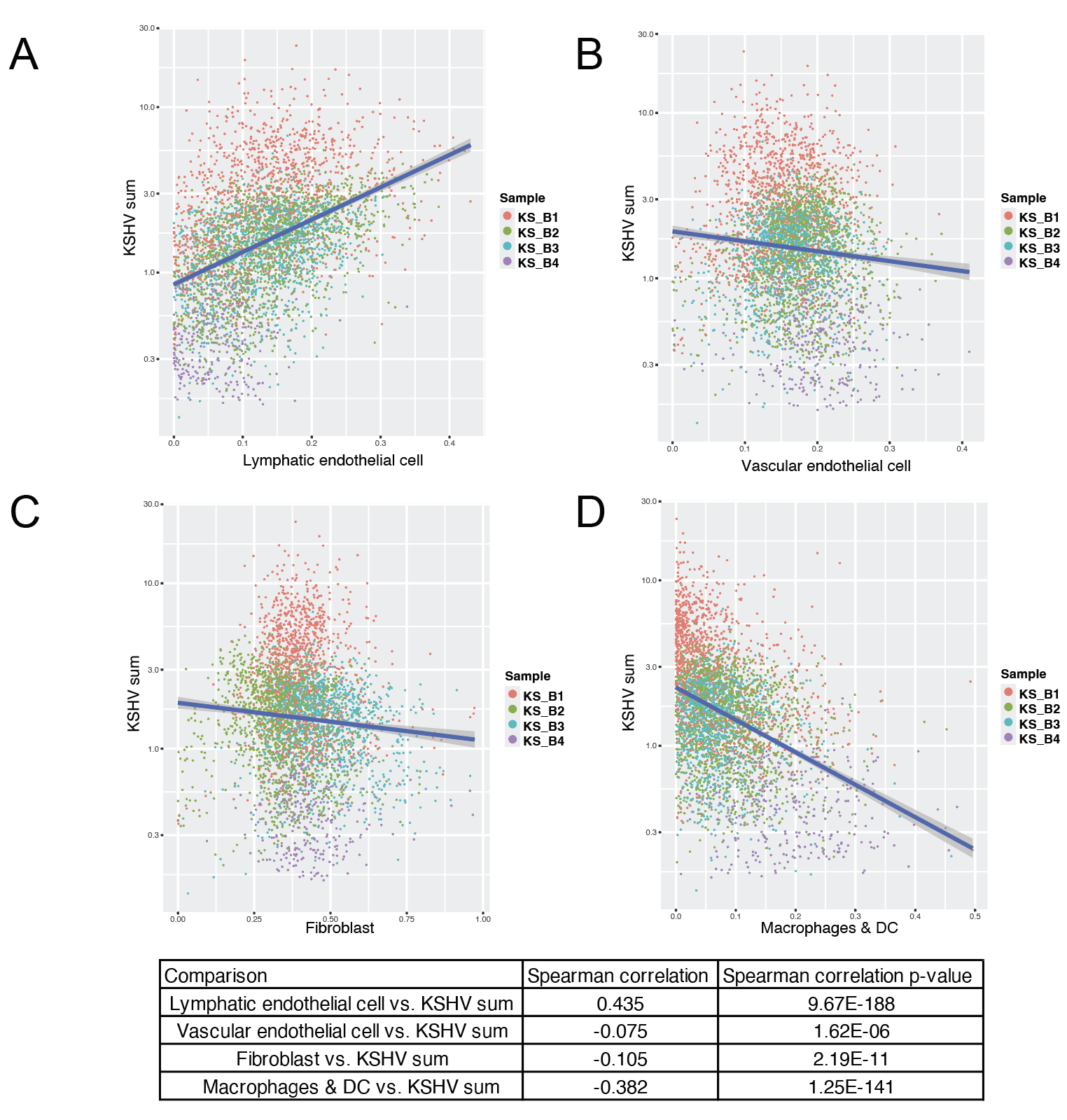


Figure S11. CARD comparison of Macrophages/DC, Lymphatic Endothelial, and Vascular Endothelial. Similar to RCDT deconvolution, KS-infected spots have much higher predicted proportions of the lymphatic endothelial cell type.

Figure S12.


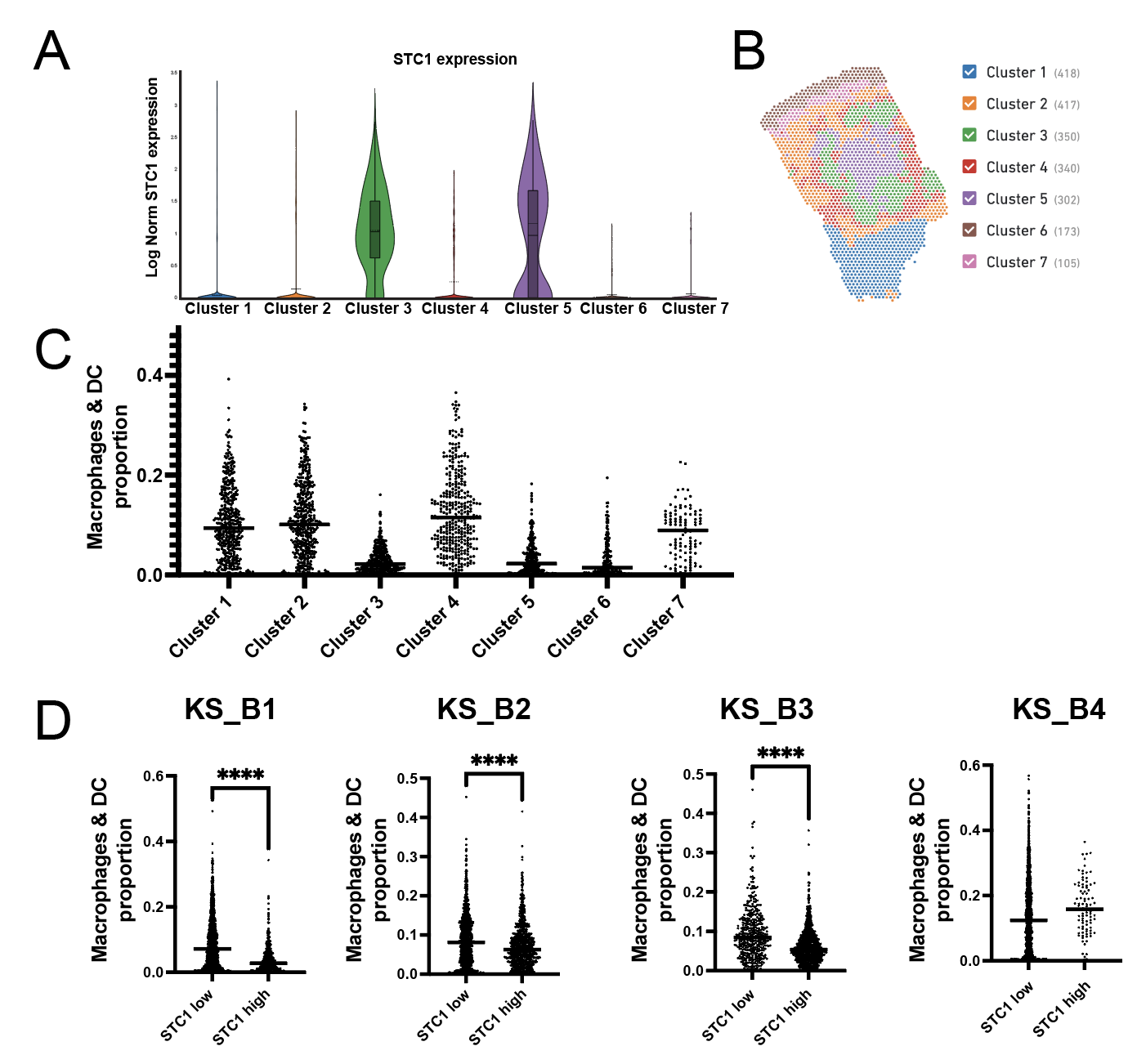


Figure S12: Expression patterns of STC1 and macrophage & dendritic cell markers using CARD deconvolution.

S10A. Violin plot of *STC1* expression in the seven clusters depicted in Figure 1.

S10B. Seven gene expression clusters in KS sample, KS_B1, are shown.

S10C. CARD-based cell-type prediction proportions for macrophages/dendritic cells for the seven clusters are plotted.

S10D. Spots for four KS samples were separated into either STC1 low or STC1 high groups. CARD-based cell-type prediction proportions for macrophages/dendritic cells are plotted on the vertical axis. Mann-Whitney test was used to test for significance, **** indicates p < 0.0001.

Figure S13

Figure S13: Spatial patterns of KSHV, *STC1*, and macrophage marker (M1, M2) expression using CARD (Similar to RCTD).

S12A. Combined expression of KSHV genes are shown in spatial plots.

S12B. Expression of *STC1* is shown in spatial plots.

S12C. CARD analysis of relative expression of M2 macrophage markers is shown.

S12D. CARD analysis of relative expression of M1 macrophage markers is shown

S12E. CARD analysis of relative expression of LEC markers is shown. As other supplementary figures show, CARD deconvolution assigns overall less proportions to LECs vs RCTD deconvolution.

Figure S14


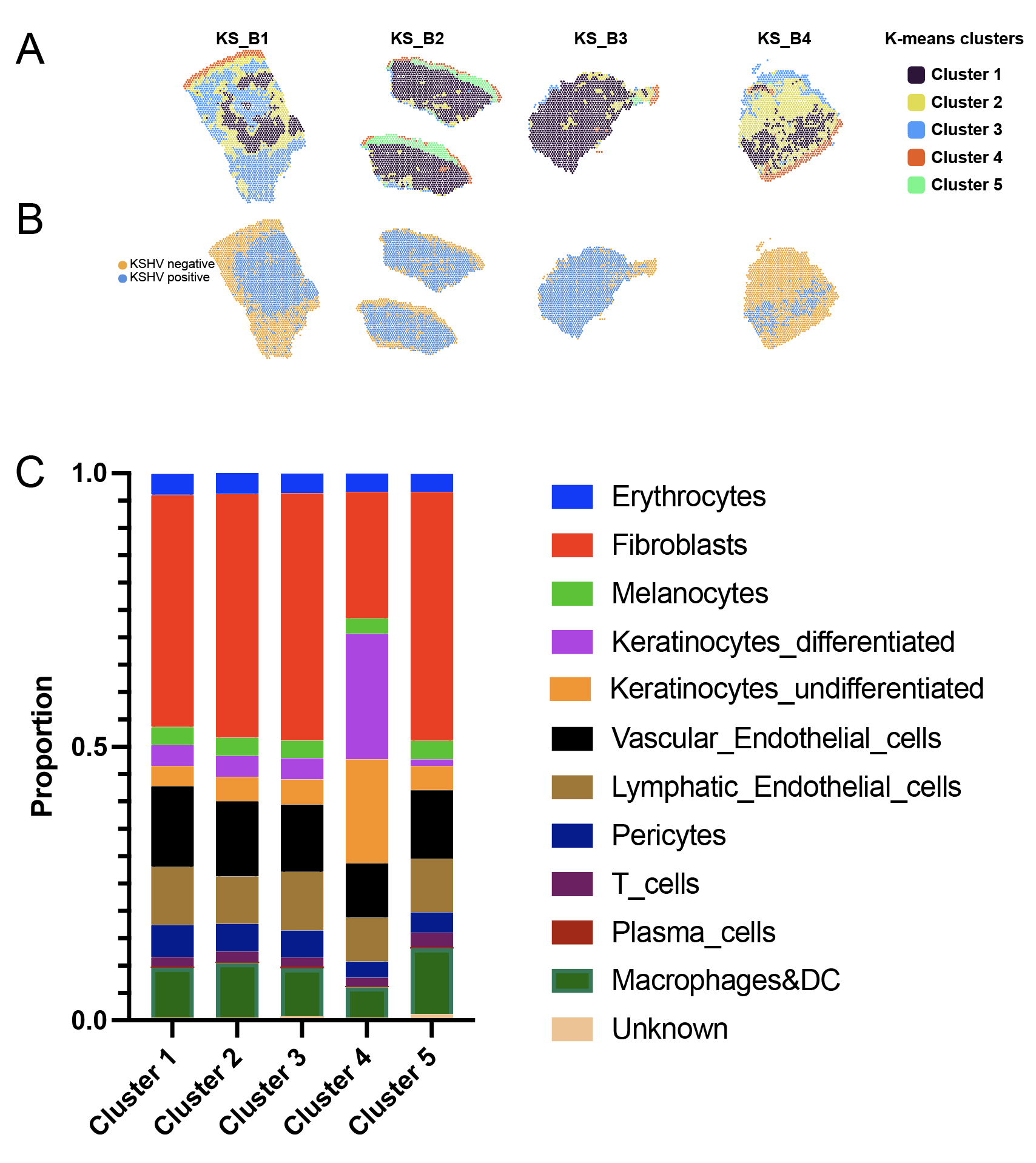


Fig S14: Integration of K-means clustering and CARD cell type predictions.

S13A-B. The same spatial plots from Fig. 2.

S13C. Relative proportions of predicted cell types from CARD analysis with the five K-means clusters.

Figure S15

Fig S15: Graph-based clustering for CARD decomposition: This figure is analogous to Fig S8 when we replace RCTD deconvolution with CARD deconvolution.

Figure S16


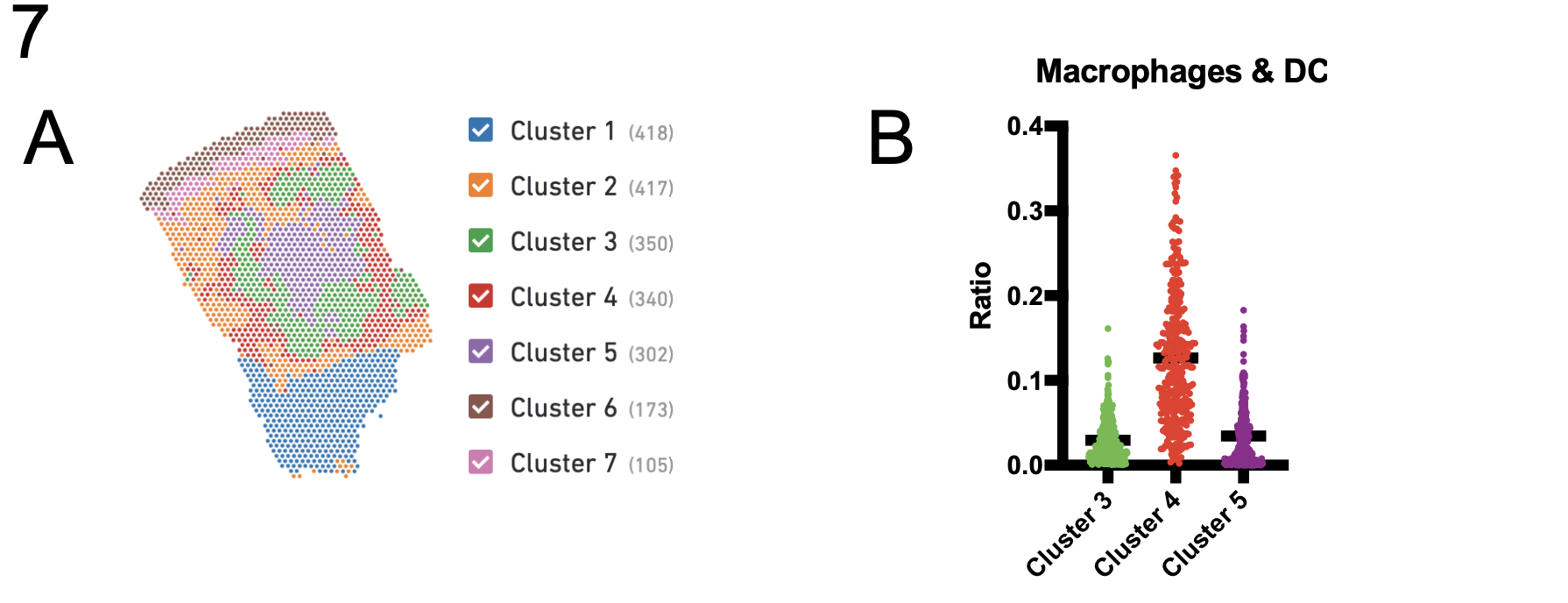


Fig S16: Markers of immune suppression surround KSHV-infected areas (Similar to Figure 7A-B).

S16A. Seven gene expression clusters in KS sample, KS_B1, are shown.

S16B. CARD-based cell-type prediction scores for macrophages/dendritic cells are shown for KSHV-infected clusters (3 and 5) and the surrounding Cluster 4.

Figure S17

|  |  |  |  |  |  |
| --- | --- | --- | --- | --- | --- |
|  |  |  |  |  |  |
|  | \|  \|  \|  \|  \|  \|  \| \| --- \| --- \| --- \| --- \| --- \| --- \| \|  \|  \|  \| 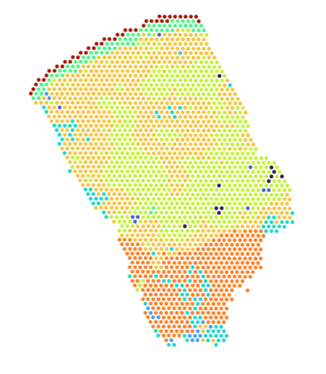 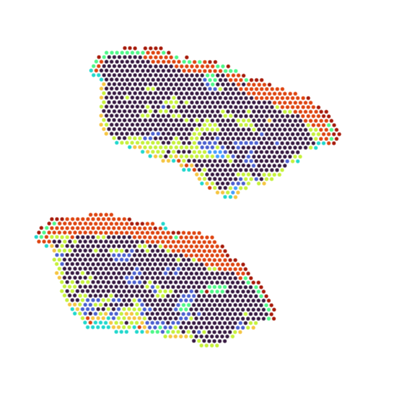  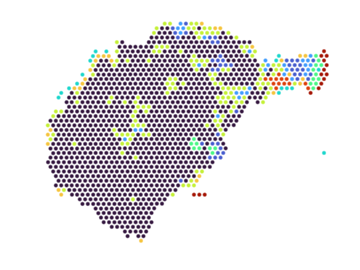 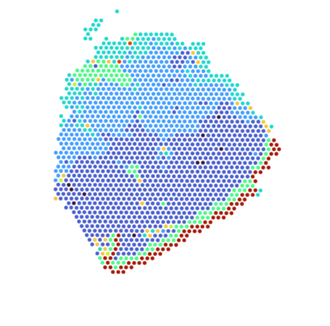 \|  \|  \| \|  \| 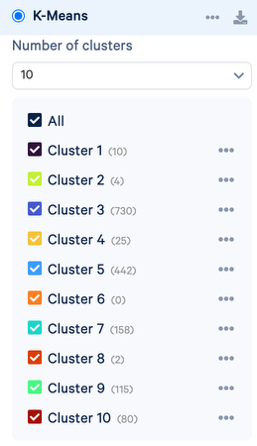 \|  \|  \|  \| \|  \|  \|  \| 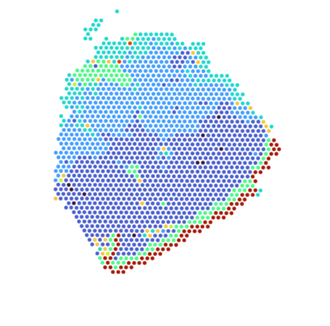 \| \|  \|  \|  \|  \| \|  \|  \|  \|  \|  \| |  |  |  |  |
|  |  |  |  |  | 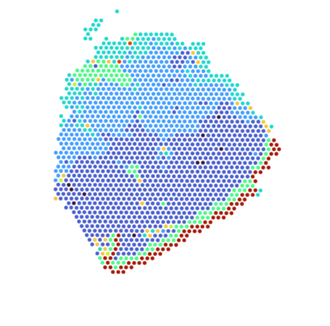 |
|  |  |  |  |  |  |
|  |  |  |  |  |  |

Fig S17: K-means cluster analysis of 4 aggregated samples (Similar to Fig. S3A). Cluster analysis of these four KS samples of 10 clusters of overall gene expression patterns. Compared to Fig S3A, this clustering seems to delineate the epidermis better Graph-based algorithm but less delineation of tumors layers.

**Supplemental Methods**

Custom viral probes for detecting KSHV genes

| **Probe name** | **Probe sequence** |
| --- | --- |
| LHS\|NC_009333_1_28819_29895\|T1_1\|1a | CCT TGG CAC CCG AGA ATT CCA CGA ATG AGC AGA TAG GTA GTG CAC C |
| LHS\|NC_009333_1_28819_29895\|T1_1\|1b | CCT TGG CAC CCG AGA ATT CCA CTA CAA CTG GCC TGG AGA TTG AAT C |
| LHS\|NC_009333_1_28819_29895\|T1_1\|1c | CCT TGG CAC CCG AGA ATT CCA GAT TGT CAC ATT TAG GGC AAA GTG G |
| LHS\|NC_009333_1_76014_76794\|K8_1\|2a | CCT TGG CAC CCG AGA ATT CCA GTA TCT TCA ACA GAT ACG GAG ACA T |
| LHS\|NC_009333_1_76014_76794\|K8_1\|2b | CCT TGG CAC CCG AGA ATT CCA CTG ATA TTA AGG CAT CGG TCA GTT C |
| LHS\|NC_009333_1_76014_76794\|K8_1\|2c | CCT TGG CAC CCG AGA ATT CCA CGA CGG TTT ACC CAA TTG CGA GGG A |
| LHS\|NC_009333_1_c118233_117535\|K12\|3a | CCT TGG CAC CCG AGA ATT CCA CGC CAA CAG ACA AAC GAG TGG TGG T |
| LHS\|NC_009333_1_c118233_117535\|K12\|3b | CCT TGG CAC CCG AGA ATT CCA CTT CAG CAT TGT ATG TGT TTA ATG T |
| LHS\|NC_009333_1_c118233_117535\|K12\|3c | CCT TGG CAC CCG AGA ATT CCA CGG ACG CCA TTT GCG TGC AAA ATG T |
| LHS\|NC_009333_1_c123815_123010\|ORF72\|4a | CCT TGG CAC CCG AGA ATT CCA GGT TGT ATT CCT GGC AAA CTG AAA A |
| LHS\|NC_009333_1_c123815_123010\|ORF72\|4b | CCT TGG CAC CCG AGA ATT CCA GGC ATA GCA AAG TGA ACT GGT AGA A |
| LHS\|NC_009333_1_c123815_123010\|ORF72\|4c | CCT TGG CAC CCG AGA ATT CCA AAG TAA CAA GAA GGA AGT GAC GTC C |
| LHS\|NC_009333_1_c134589_130655\|ORF75\|5a | CCT TGG CAC CCG AGA ATT CCA CTA GCA GGG TCT GTG TGA TTT TGG A |
| LHS\|NC_009333_1_c134589_130655\|ORF75\|5b | CCT TGG CAC CCG AGA ATT CCA GGT CTG TCC GCC TGG AAA CAG GGT G |
| LHS\|NC_009333_1_c134589_130655\|ORF75\|5c | CCT TGG CAC CCG AGA ATT CCA GTA GTG GTA GAG ATG GAG CAG ACT G |
| LHS\|NC_009333_1_c134589_130655\|ORF75\|5d | CCT TGG CAC CCG AGA ATT CCA CAG GTC GTC TAC TAT TCT GAT GCC T |
| RHS\|NC_009333_1_28819_29895\|T1_1\|1a | /5Phos/AC TGT TCT GAT ACA CCA GTG GGC GC AA AAA AAA AAA AAA AAA AAA AAA AAA AAA A |
| RHS\|NC_009333_1_28819_29895\|T1_1\|1b | /5Phos/ CA ATG CAA TAA CCC GCA AGG GGT GA AA AAA AAA AAA AAA AAA AAA AAA AAA AAA A |
| RHS\|NC_009333_1_28819_29895\|T1_1\|1c | /5Phos/ CC CGA TTT ACA CTC AAT CCG CTT TC AA AAA AAA AAA AAA AAA AAA AAA AAA AAA A |
| RHS\|NC_009333_1_76014_76794\|K8_1\|2a | /5Phos/ TT GGA TAC TCA CTC GAC GGA GAT CC AA AAA AAA AAA AAA AAA AAA AAA AAA AAA A |
| RHS\|NC_009333_1_76014_76794\|K8_1\|2b | /5Phos/ TG TGG TGG CCT GGA CGC TTT GTG TC AA AAA AAA AAA AAA AAA AAA AAA AAA AAA A |
| RHS\|NC_009333_1_76014_76794\|K8_1\|2c | /5Phos/ TT ACA GTT GAA ACG TGT AAT ACA CC AA AAA AAA AAA AAA AAA AAA AAA AAA AAA A |
| RHS\|NC_009333_1_c118233_117535\|K12\|3a | /5Phos/ AT CGC CCC AAG CCT CCA GCG CCA CC AA AAA AAA AAA AAA AAA AAA AAA AAA AAA A |
| RHS\|NC_009333_1_c118233_117535\|K12\|3b | /5Phos/ TG TGC TTG GTG CAA CCG TGA TTG TG AA AAA AAA AAA AAA AAA AAA AAA AAA AAA A |
| RHS\|NC_009333_1_c118233_117535\|K12\|3c | /5Phos/ GC TTT GCT GGA GGC CAA CTT CCG TC AA AAA AAA AAA AAA AAA AAA AAA AAA AAA A |
| RHS\|NC_009333_1_c123815_123010\|ORF72\|4a | /5Phos/ CA TCC ATG TGC CCA GTA ACT TAC GC AA AAA AAA AAA AAA AAA AAA AAA AAA AAA A |
| RHS\|NC_009333_1_c123815_123010\|ORF72\|4b | /5Phos/ AT AGG CGT GAG GCT TCT GAG CTT AC AA AAA AAA AAA AAA AAA AAA AAA AAA AAA A |
| RHS\|NC_009333_1_c123815_123010\|ORF72\|4c | /5Phos/ GT CGC TAA GAC TGC CTC TGT TCG CC AA AAA AAA AAA AAA AAA AAA AAA AAA AAA A |
| RHS\|NC_009333_1_c134589_130655\|ORF75\|5a | /5Phos/ CA CCA GGT GCC TGC CCA CTT CCA CT AA AAA AAA AAA AAA AAA AAA AAA AAA AAA A |
| RHS\|NC_009333_1_c134589_130655\|ORF75\|5b | /5Phos/ CT GTG AAA CAA CAG GTT GCC AAG GC AA AAA AAA AAA AAA AAA AAA AAA AAA AAA A |
| RHS\|NC_009333_1_c134589_130655\|ORF75\|5c | /5Phos/ CC AGG GCG TTG CCA GGA GTG GTG GC AA AAA AAA AAA AAA AAA AAA AAA AAA AAA A |
| RHS\|NC_009333_1_c134589_130655\|ORF75\|5d | /5Phos/ GG CTG CTC CGA GGT CCT CCC GAG GA AA AAA AAA AAA AAA AAA AAA AAA AAA AAA A |

Graph-based Algorithm of the Manufacturer (10X Genomics) implemented in Loupe-Browser

We used the default Loupe Browser v7 from 10X Genomics. The default algorithm is a graph-based clustering algorithm that involves building a sparse k-nearest-neighbor graph inspired by the Louvain Modularity Optimization [1]. The spots are represented in their top Principal Component to reduce the noise, and the distance (dissimilarity) between the spots is defined based on the Euclidean distance in this representation. The algorithm aims to identify highly connected “modules” within the graph. The value of k, which represents the number of nearest neighbors, is set to be logarithmic to the number of spots. Moreover, a hierarchical clustering step is performed to merge the smaller clusters.  If two siblings in the hierarchical clustering have no differentially expressed genes (with FDR p-value < 0.05), they are merged into a single cluster. This process is repeated until no merging is required.

Predictor (classifier)

We looked for a human gene signature for virally infected spots in these KS tissues. We deemed any spot with at least one transcript of KSHV genes to be infected. We performed a cross-sample validation to validate our signature, i.e., we left out one of the four KS samples with KS probes as the test sample and one of the six normal samples. We traversed all 24 possible combinations and augmented their predictions on the unseen samples. This validation process was designed to prevent introducing sample bias in the validation of the prediction. We plotted the Receiver Operating Characteristic (ROC) curve, which showed 0.96 area under the curve.

For preprocessing, we used the Seurat package (v. 5.1.0) pipeline in R [2]. We merged 10 samples using the merge function, followed by NormalizData, FindVariable, and ScaleData. To remove the bias, we applied the Harmony package (v. 1.2.1) [3]. We used the scaled data for prediction.

For building the classifier, we built a Logistic Regression with L1 penalty on differentially expressed genes. For differentially expressed genes, we used genes whose t-test effect size is greater than the median (Cohen’s d > 0.5 [4]). We did not use p-value, due to the abundance of spots, not many genes would be filtered. Thereafter, we built a Logistic Regression with L1 penalty and applied an inner loop of cross-validation on the training samples to find the optimized value for lasso penalty value, lambda (lambda.1se from the output of the function cv.glmnet from package glmnet 4.1.8 with default arguments [5]). Finally, we trained the classifier using the optimized lambda on the whole training sample.

As the final signature, we built the classifier using our method with all 10 samples to report as our main signature for KS-positive spots. To determine which gene is more important in the signature, we calculated the feature importance score as the following: the coefficient multiplied by the average difference between KS+ and KS-, as reported in Fig 3B, along with the averages.

Cell Type Deconvolution Analysis

Even though the Spatial 3’ v1 or v2 chemistry Visium platform has a relatively high resolution, it is not yet at the single-cell resolution. Hence, we used RCTD as our main deconvolution algorithm [6], as well as CARD Analysis as an alternative algorithm [7], both spatially aware cell deconvolution for spatial transcriptome data. In short, RCTD and CARD need an annotated single-cell reference, and they deconvolve each spot based on that reference into different cell types. Furthermore, CARD refines the mapping using a spatially aware Auto-Regressive filter, which makes the outcome spatially coherent compared to a spatially agnostic algorithm. Notably, CARD analysis uses whole annotated single cells, and the algorithm identifies informative genes agnostically and in a multi-variable analysis of these informative genes for cell deconvolution. For the majority of our analysis, we utilized GSE130973 single-cell data, which encompassed 11 distinct cell types from healthy skin. For more specific immune cell typing, we used another data set GSE214695. We reported the differentially expressed genes to distinguish between M1 and M2 using Seurat FindMarkers with default parameters. The data can be found as a supplement file (Table S1). We did not combine both cell references to avoid introducing batch and experiment effects in our analysis. Thus, the M2 and M1 proportions must be considered a relative deconvolution, not an absolute number.

 References

1. Blondel, V. D., Guillaume, J. L., Lambiotte, R., & Lefebvre, E. (2008). Fast unfolding of communities in large networks. Journal of statistical mechanics: theory and experiment, 2008(10), P10008.
2. Hao, Y., Stuart, T., Kowalski, M. H., Choudhary, S., Hoffman, P., Hartman, A., ... & Satija, R. (2024). Dictionary learning for integrative, multimodal and scalable single-cell analysis. *Nature biotechnology*, *42*(2), 293-304
3. Korsunsky, I., Millard, N., Fan, J., Slowikowski, K., Zhang, F., Wei, K., ... & Raychaudhuri, S. (2019). Fast, sensitive and accurate integration of single-cell data with Harmony. *Nature methods*, *16*(12), 1289-1296.
4. Cohen, J. (2013). *Statistical power analysis for the behavioral sciences*. routledge.
5. Friedman, J. H., Hastie, T., & Tibshirani, R. (2010). Regularization paths for generalized linear models via coordinate descent. *Journal of statistical software*, *33*, 1-22.
6. Cable DM, Murray E, Zou LS, Goeva A, Macosko EZ, Chen F, Irizarry RA. Robust decomposition of cell type mixtures in spatial transcriptomics. Nature biotechnology. 2022 Apr;40(4):517-26.
7. Ma, Y., Zhou, X. Spatially informed cell-type deconvolution for spatial transcriptomics. Nat Biotechnol 40, 1349–1359 (2022).
